# Significance of methylation-related genes in diagnosis and subtype classification of renal interstitial fibrosis

**DOI:** 10.1186/s41065-023-00295-8

**Published:** 2023-07-27

**Authors:** Hanchao Zhang, Yue Yang, Zhengdao Liu, Hong Xu, Han Zhu, Peirui Wang, Guobiao Liang

**Affiliations:** 1 Department of Urology, The Affilated Hospital and Clinical Medical College of Chengdu University, Chengdu, Sichuan China; 2 Medical College of Soochow University, Suzhou, Jiangsu China; 3 Department of Urology, Affiliated Hospital of Zunyi Medical University, Zunyi, Guizhou China

**Keywords:** Renal interstitial fibrosis, RNA methylation-related gene, Diagnosis, Subtype classification

## Abstract

**Background:**

RNA methylation modifications, such as N1-methyladenosine/N6-methyladenosine /N5-methylcytosine (m^1^A/m^6^A/m^5^C), are the most common RNA modifications and are crucial for a number of biological processes. Nonetheless, the role of RNA methylation modifications of m^1^A/m^6^A/m^5^C in the pathogenesis of renal interstitial fibrosis (RIF) remains incompletely understood.

**Methods:**

Firstly, we downloaded 2 expression datasets from the GEO database, namely GSE22459 and GSE76882. In a differential analysis of these datasets between patients with and without RIF, we selected 33 methylation-related genes (MRGs). We then applied a PPI network, LASSO analysis, SVM-RFE algorithm, and RF algorithm to identify key MRGs.

**Results:**

We eventually obtained five candidate MRGs (WTAP, ALKBH5, YTHDF2, RBMX, and ELAVL1) to forecast the risk of RIF. We created a nomogram model derived from five key MRGs, which revealed that the nomogram model may be advantageous to patients. Based on the selected five significant MRGs, patients with RIF were classified into two MRG patterns using consensus clustering, and the correlation between the five MRGs, the two MRG patterns, and the genetic pattern with immune cell infiltration was shown. Moreover, we conducted GO and KEGG analyses on 768 DEGs between MRG clusters A and B to look into their different involvement in RIF. To measure the MRG patterns, a PCA algorithm was developed to determine MRG scores for each sample. The MRG scores of the patients in cluster B were higher than those in cluster A.

**Conclusions:**

Ultimately, we concluded that cluster A in the two MRG patterns identified on these five key m^1^A/m^6^A/m^5^C regulators may be associated with RIF.

**Supplementary Information:**

The online version contains supplementary material available at 10.1186/s41065-023-00295-8.

## Introduction

Low public awareness, high prevalence, high medical expenses, and a poor prognosis are all features of the significant public health threat known as chronic kidney disease (CKD), which affects people all over the world [1]. 697.5 million individuals worldwide, or 9.1% of the population, had CKD in 2017 [2]. Renal interstitial fibrosis (RIF) is a common pathological response to the widespread incidence of CKD. RIF is defined by the abnormal accumulation of extracellular matrix in the interstitial compartment situated between the tubules and peritubular capillaries, leading to tissue damage and functional impairment. Ultimately, this accumulation of matrix results in renal failure and eventual mortality [3, 4]. Unfortunately, there are no targeted, definitive, and effective treatments for RIF, so early recognition of patients at high risk for RIF is above all important for early prevention, delay, and reversal of the disease.

RNA modifications that occur in cells play an important role in regulating their stability, processing, transport, and gene expression and are the central switch in RNA metabolism [5]. RNA methylation, as the most typical modification in RNA modification, can be classified into various forms, including m^1^A, m^6^A and m^5^C, depending on the methylation site [6, 7]. Similar to histone modifications and DNA epigenetics, RNA methylation modifications can be installed, removed, and recognized by specific proteins called “writers,“ “erasers,“ and “readers”. The methylation modification known as m^6^A is formed by methylation of the sixth N atom of RNA adenine with the assistance of several m^6^A writers, including METTL3, METTL14, and WTAP [8–10]. Conversely, demethyltransferases (FTO and ALKBH5) that remove m^6^A, called m^6^A erasers, work with m^6^A writers to maintain a dynamic balance between mRNA methylation and unmethylation in cells [11]. YTHDF1/2/3 and YTHDC1/2 (YTH structural domain family proteins), as a category of RNA-binding proteins, recognize m^6^A modification sites that lead to changes in RNA metabolism [12, 13]. Under healthy circumstances, adenine is a positively electrostatically methylated nucleotide, and the m^1^A modification is a reversible methylation modification of the first nitrogen (N1) atom of adenine in RNA [14].

Similar to m^6^A, the m^1^A modification process is also regulated by m^1^A writers, erasers, and readers [11]. The m^5^C is a methylation alteration on the fifth carbon atom of cytosine that is extensively prevalent in a number of RNAs, including mRNA, tRNA, rRNA, and lncRNA. More than 10 RNA m^5^C methyltransferases have been discovered so far, including DNMT2, NSUN, and TRDMT [15]. The m^5^C site is primarily recognized by the reader proteins YBX1 and ALYREF [16, 17]. Erasers of m^5^A have not been identified, with only partial reports suggesting that the TET family has the potential to function as RNA demethylases [18]. In conclusion, the most common RNA methylation modifications have their own unique regulators and complex regulatory mechanisms, and they play a crucial role in living organisms. Abnormal RNA modification can promote the occurrence and development of various tumors by regulating cell growth, differentiation, migration, and drug resistance, such as gastric cancer, liver cancer, colorectal cancer, etc. [11].

The alteration of the apparent transcriptional profile of m^6^A in UUO mouse’s kidney signifies the crucial role played by m^6^A in the regulation of renal interstitial RIF [19]. In their study, Cui and colleagues utilized m^6^A methylation profiling to elucidate the role of m^6^A methylation in modulating oxidative stress and cytoplasmic metabolism during the development of liver fibrosis. Notably, their findings revealed distinct patterns of m^6^A methylation that were enriched in immune response and apoptosis, which were associated with fibrosis regression [20]. Moreover, Li et al. discovered that the activation of cardiac fibroblasts is closely associated with elevated expression of METTL3. Cui and colleagues utilized m^6^A methylation profiling to elucidate the role of m^6^A methylation in modulating oxidative stress and cytoplasmic metabolism during the development of liver fibrosis [21]. Notably, their findings revealed distinct patterns of m^6^A methylation that were enriched in immune response and apoptosis, which were associated with fibrosis regression [22].Unfortunately, the function of the m^1^A/m^6^A/m^5^C regulators in RIF is still unknown.

In the present study, we examined the roles of m^1^A/m^6^A/m^5^C regulators in the diagnosis and subtype classification of RIF using the GSE22459 and GSE76882 datasets in the GEO. We are based on five key m^1^A/m^6^A/m^5^C regulators (ALKBH5, ELAVL1, RBMX, WTAP, and YTHDF2). A genetic model for predicting RIF susceptibility was established, and patients were shown to derive good clinical benefit from this model. Furthermore, we constructed two distinct MRG models that closely correlate with the expression of genes implicated in RIF, suggesting that MRGs hold promise as diagnostic and subtype classification markers for RIF, with potential implications for the early intervention and treatment of this disease.

## Materials and methods

### Acquisition of data

The GSE22459 and GSE76882 datasets from the GEO database contain 99 healthy patients without RIF and 135 patients with RIF [23, 24]. In light of the existing literature, we have identified a total of 48 genes responsible for regulating m^1^A, m^6^A, and m^5^C modifications (**Supplementary Table 1**) [25–27]. We employed a two-fold approach to preprocessing our data. Firstly, we applied a log2 transformation to each dataset and normalized the resulting expression values separately. Secondly, we merged the two datasets and utilized the “ComBat” function, a popular batch correction algorithm, to eliminate the batch effect. This process ensured that any system-specific variations between the two datasets were effectively removed, resulting in a more robust and reliable dataset for downstream analysis [28]. To identify the MRGs associated with RIF, we conducted differential analysis between patients with and without RIF. Specifically, we utilized the R package “limma” to analyze the differentially expressed MRGs between the two groups, adopting the Wilcoxon test with a significance threshold of p value less than 0.05.

### Identification of key MRGs

To model RIF, we applied the protein-protein interaction (PPI) network, least absolute shrinkage and selection operator (LASSO) analysis, support vector machine recursive feature elimination (SVM-RFE) algorithm, and random forest (RF) algorithm to identify key MRGs. To establish the PPI network, we initially imported 48 genes previously identified as m^1^A/m^6^A/m^5^C regulators into the STRING protein database. We subsequently applied a minimum interaction score threshold of 0.4, followed by utilization of cytoscape and the MCODE algorithm to identify the central network and hub genes [29, 30]. A higher interaction score indicated a more definite protein-protein relationship; an interaction score of 0.4 was the default parameter in the STRING database, and most previous research had been screened using this as the threshold [31, 32]. The LASSO analysis is a dimensionality reduction algorithm that exhibits superior performance compared to regression analysis [33]. We used a 10-fold cross-validation lasso analysis to screen for hub genes. The SVM-RFE algorithm is a machine learning technique that involves the use of SVMs for generating feature vectors and iteratively eliminating them to identify the optimal set of variables [34]. The RF algorithm is a component-based supervised learning method, often viewed as an extension of decision trees [35]. We used the “random forest” package in R software to build a RF model (ntree = 500) to select key regulator genes from differentially expressed MRGs. Finally, the hub MRGs were identified by taking the intersection of the four analysis methods.

### Establishment of a nomogram model

We developed a nomogram model utilizing five candidate MRGs and utilized the “rms” package in R to predict the incidence of interstitial fibrosis in patients. Furthermore, we created calibration curves to examine the agreement between projected and actual results. Finally, to determine whether the model would benefit patients, we ran a decision curve analysis (DCA) and exhibited clinical effect curves [36, 37].

### Identification of molecular subtype

To gain deeper insights into the molecular mechanisms of RIF, we employed the consensus clustering algorithm, a powerful tool that identifies subgroups of similar patterns within a given dataset. Utilizing the R package “Consensus Cluster Plus”, we conducted unsupervised clustering analysis to identify the distinct patterns of MRGs based on their expression profiles. Through the use of resampling techniques, we were able to validate the clusters generated and establish the robustness of our analyses [38]. The selection of the number of clusters involves consideration of three primary criteria: small intra-group differences, large inter-group differences, and sufficient sample sizes. We employed various methods, including consensus matrix plots, consensus cumulative distribution function (CDF) plots, relative changes in area under the CDF curve, and trace plots, to determine the optimal number of clusters. Subsequently, we generated histograms to visualize the expression differences in the five significant MRGs between the two MRG clusters.

### Estimation of the genetic profile

In order to quantify the MRG patterns identified from our clustering analysis, we employed the principal component analysis (PCA) algorithm to calculate the MRG scores for each RIF sample. Specifically, we used the first and second principal components (PC1 and PC2, respectively) as representative scores for each sample. To compute an MRG score for each RIF patient, we used the following equation: MRG Score = Σ(PC1i + PC2i), where i represents the expression values of MRGs. This allowed us to generate a comprehensive score index that represented the MRG expression patterns for each RIF patient, providing a quantitative basis for subsequent analyses and interpretations.

### Estimation of the immune cell infiltration

Single-sample gene set enrichment analysis (ssGSEA) has been widely used in the field of immunological research to quantify the relative abundance of specific immune cell populations present within complex biological samples. As such, ssGSEA has emerged as a powerful tool for evaluating the immune microenvironment in various disease states, including RIF. By analyzing gene expression data obtained from RIF patient samples, we were able to utilize ssGSEA to establish the abundance of immune cells present within each sample [39]. This information may contribute towards a better understanding of the role that immune cells play in driving RIF pathogenesis, and could potentially inform the development of novel immunotherapeutic approaches for the treatment of RIF.

### Functional and pathway enrichment analysis

To further investigate the biological significance of our findings, we utilized a number of R packages, including “clusterProfiler”, “ComplexHeatmap”, “limma”, “colorspace”, “stringi”, and “ggplot2”, to conduct GO and KEGG enrichment analyses. These analyses allowed us to identify the functional and molecular pathways associated with the differentially expressed genes (DEGs) between the two MRG clusters (A and B) we identified (adjusted P value < 0.05) [40, 41]. By examining the enriched pathways and functions, we were able to derive a richer understanding of the underlying biological mechanisms and potential therapeutic targets relevant to RIF.

## Results

### Landscape of the 48 m1A/m6A/m5C regulators

**Fig. 1:**
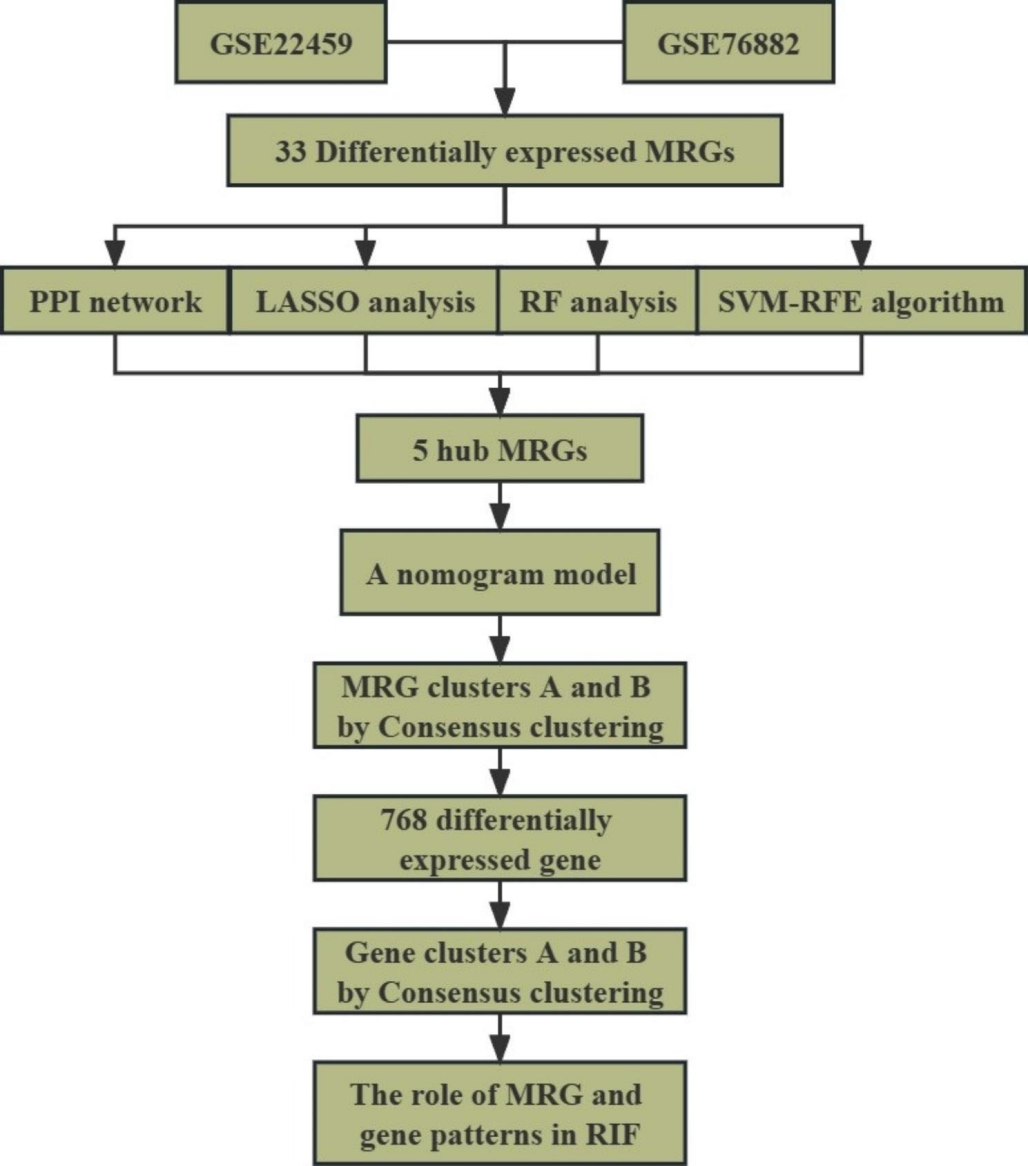
The research flow

**Fig. 2:**
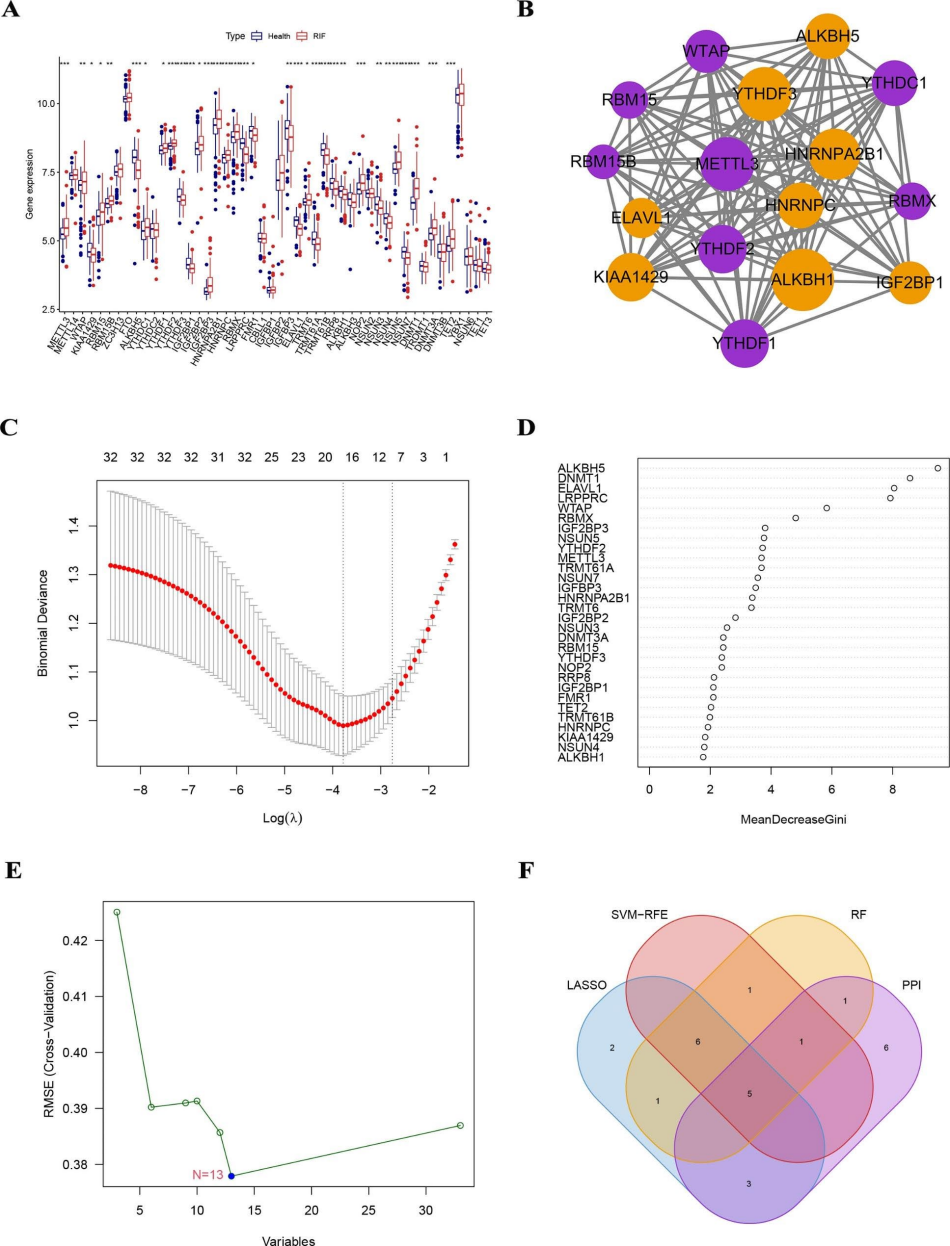
Identification of five key MRGs. (**A**) Landscape of the 48 MRGs in RIF. (**B**) 16 MRGs obtained from the PPI network. (**C**) 17 MRGs were identified by the LASSO analysis. (**D**) 15 MRGs were identified by the RF model. (**E**) Thirteen MRGs were selected by the SVM-RFE algorithm. (**F**) Five key MRGs (ALKBH5, ELAVL1, RBMX, WTAP, and YTHDF2) were finally obtained after applying four algorithm

The present study depicts its research methodology in Fig. 1, which showcases the research flow. Additionally, Table 1 offers an overview of the primary R packages utilized in this study and the functions they implement. To examine the differential expression levels of 48 MRGs within patients with and without RIF, we employed the “limma” package within R followed by the generation of histograms to display the results. We discovered 33 differentially expressed MRGs, in which METTL3, WTAP, RBMX, YTHDF2, DNMT1, and other regulatory factors were over-expressed in patients with RIF, whereas ALKBH5, ELAVL1, TRMT61B, and other regulatory factors were down-regulated (Fig. 2A **and Supplementary Table 2**).

**Table 1:** An overview of the primary R packages utilized in this study and the functions they implement

R package	Versions	Function
sva	3.44.0	Batch correction
limma	3.52.1	Differential expression analysis
glmnet	4.1−4	LASSO analysis
randomForest	4.7–1.1	RF algorithm
caret	6.0–92	SVM-RFE algorithm
venn	1.11	Taking the intersection
rms	6.3−0	Constructing a nomogram model
ConsensusClusterPlus	1.60.0	Unsupervised clustering analysis
ggplot2	3.4.1	Drawings
clusterProfiler	4.6.1	Enrichment analysis
GSVA	1.44.2	Gene Set Variation Analysis

### Identification of of key MRGs

To further screen for MRGs significantly associated with RIF, we used the PPI network, LASSO analysis, SVM-RFE algorithm, and RF algorithm to identify key regulatory genes. Using the String database, we investigated the interaction network of 33 differentially expressed MRGs and visualized them using the cytoscape software. We obtained 16 MRGs based on interaction scores greater than 0.4 and the MCODE algorithm (Fig. 2B). Using the LASSO algorithm, we identified 17 MRGs from the 33 differentially expressed MRGs (Fig. 2C). The 15 MRGs were identified by constructing the RF model (Fig. 2D). The tenfold cross-validation curve shows that the accuracy of SVM-RFE is highest only when we select the 13 MRGs (Fig. 2E). Finally, we overlapped the results of the above three different algorithms and PPI network analysis to obtain five MRGs (ALKBH5, ELAVL1, RBMX, WTAP, and YTHDF2) that were significantly associated with RIF (Fig. 2F).

### Construction of the nomogram

**Fig. 3:**
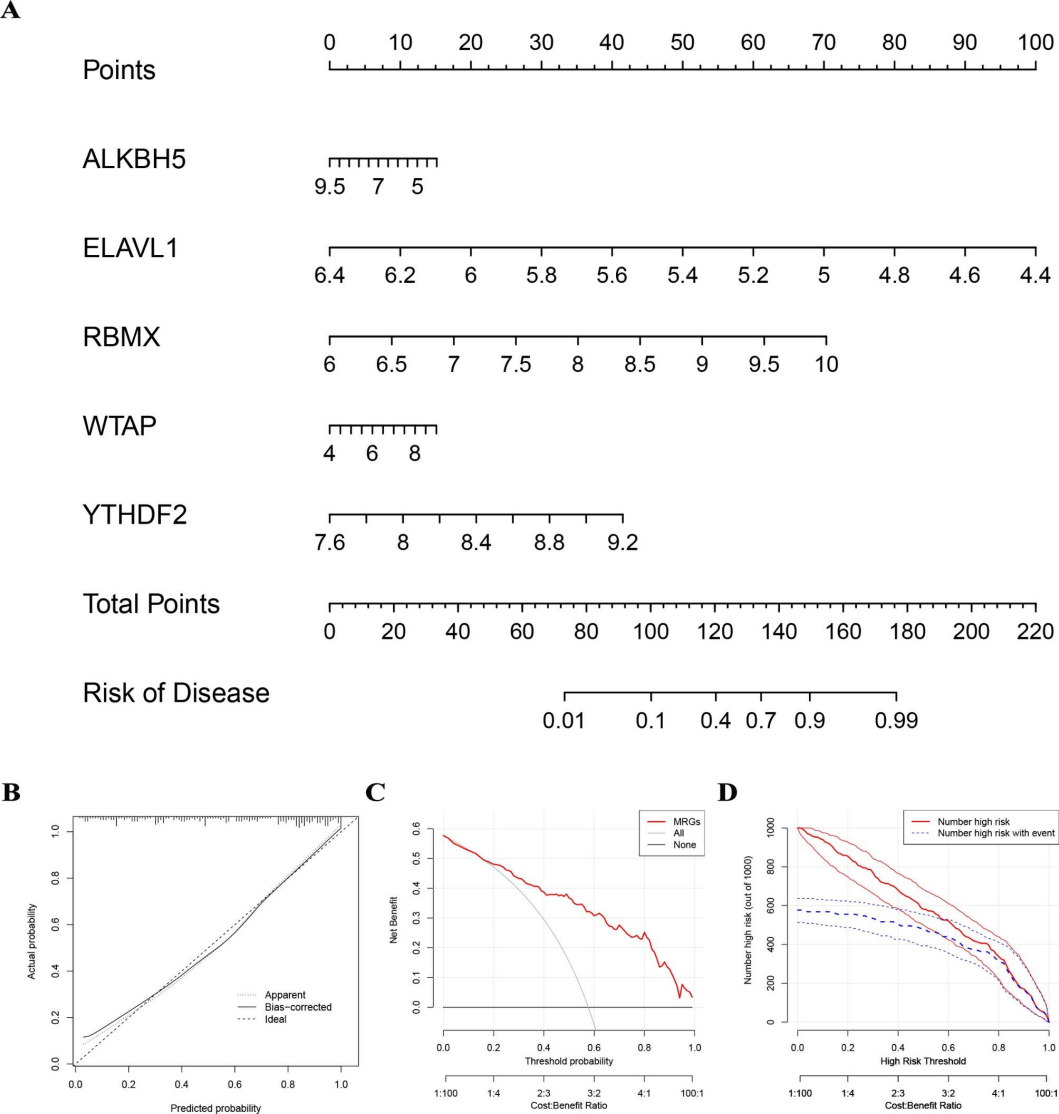
(**A**) The construction of the nomogram model according to the five candidate MRGs. (**B**) The predictive ability of the nomogram model as exposed by the calibration curve. (**C**) Decisions based on the nomogram model may benefit patients with RIF. (**D**) The clinical impact of the nomogram model as assessed by the clinical impact curve

Based on our previous selection of five candidate MRGs, we built a nomogram model to predict the incidence of RIF (Fig. 3A). The calibration curve demonstrates that the nomogram model’s predictiveness is correct (Fig. 3B). Nomogram model-based decisions hold potential advantages for RIF patients, as evidenced by the DCA curve displaying the red line remaining consistently above the gray and black lines within the range of 0 to 1 (Fig. 3C). Furthermore, the clinical impact curve shows the nomogram model to possess robust predictive power (Fig. 3D).

### Identification of two different MRG patterns

**Fig. 4:**
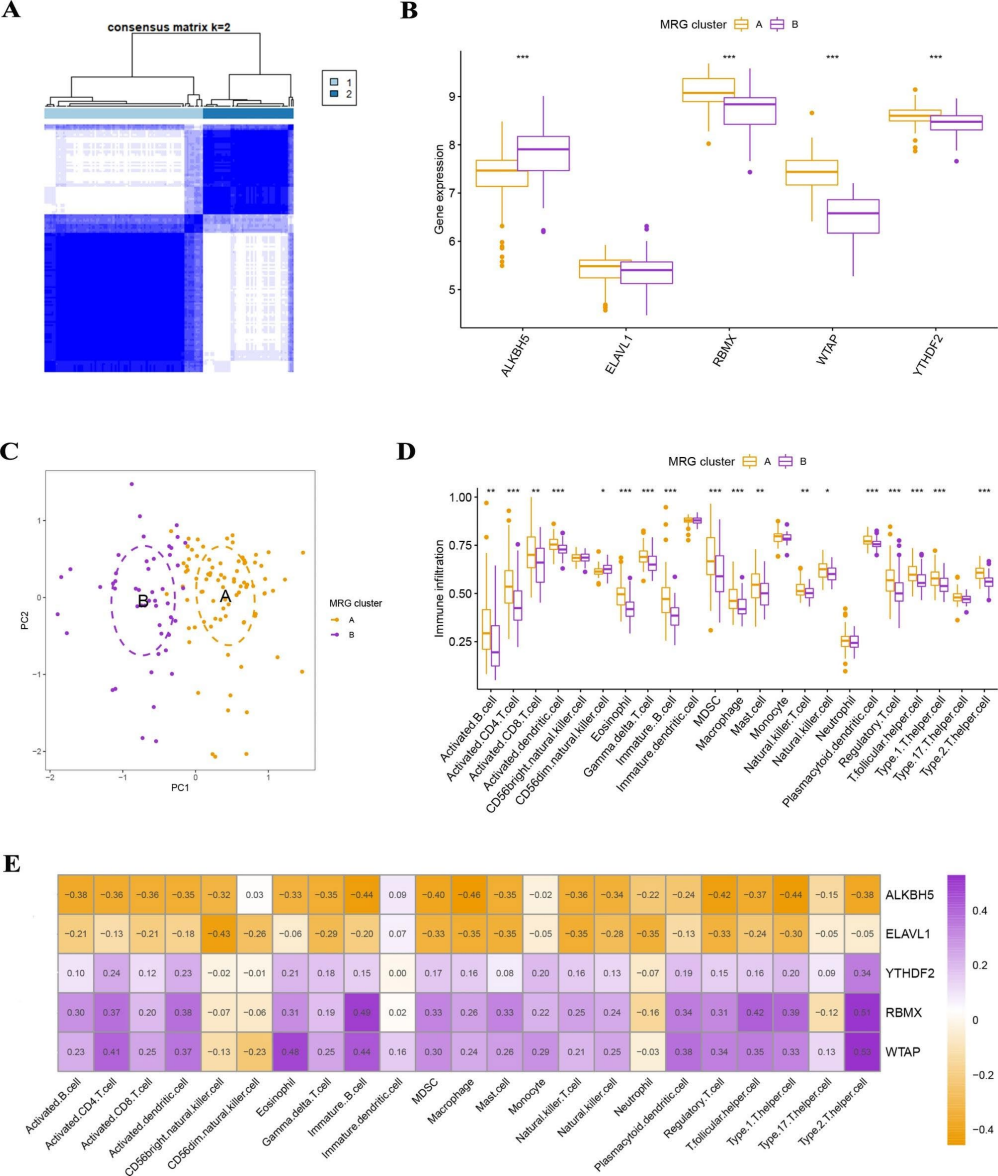
Consensus clustering of five significant MRGs. (**A**) Consensus matrices of five significant MRGs for k = 2. (**B**) A differential expression histogram of five noteworthy MRGs in MRG clusters A and B. (**C**) PCA for the expression profiles of five significant MRGs. (**D**) Differences in cell infiltration between MRG clusters A and B. (**E**) Connection between infiltrating immune cells and five significant MRGs

Based on the five significant MRGs, two MRG patterns (MRG clusters A and B) were identified by using the “ConsensusClusterPlus” package in R software and the consensus clustering method. (Fig. 4A **and Supplementary Fig. 1**). We then plotted histograms to observe the differences in the expression levels of the 5 MRGs between these two MRG clusters (Fig. 4B). RBMX, WTAP, and YTHDF2 were expressed at higher levels in MRGcluster A than in MRGcluster B, while the opposite was true for ALKBH5. ELAVL1 expression did not differ significantly between MRG clusters A and B. PCA shows that the two MRG models can be fully distinguished based on these five important MRGs (Fig. 4C). We next calculated the immune cell abundance in the RIF samples using ssGSEA, and we produced histograms to demonstrate the various immune cell infiltrations between the two MRG clusters (Fig. 4D). In MRG cluster A, we discovered that activated B cells, activated CD4 T cells, activated CD8 T cells, activated dendritic T cells, eosinophils, gamma delta T cells, immature B cells, MDSCs, macrophages, mast cells, natural killer T cells, plasmacytoid dendritic cells, regulatory T cells, T follicular helper cells, type 1 T helper cells, and type 2 T helper cells were significantly expressed (p < 0.05). In addition, we evaluated the correlation between the five MRGs and immune cells separately and plotted the histogram (Fig. 4E). We found that WTAP was positively correlated with many immune cells, except CD56 bright natural killer cells, CD56 dim natural killer cells, and neutrophils. And ALKBH5 was negatively correlated with many immune cells, except CD56 dim natural killer cells, and immature dendritic cells. We classified the five key MRGs into high and low expression groups according to their median gene expression levels.

**Fig. 5:**
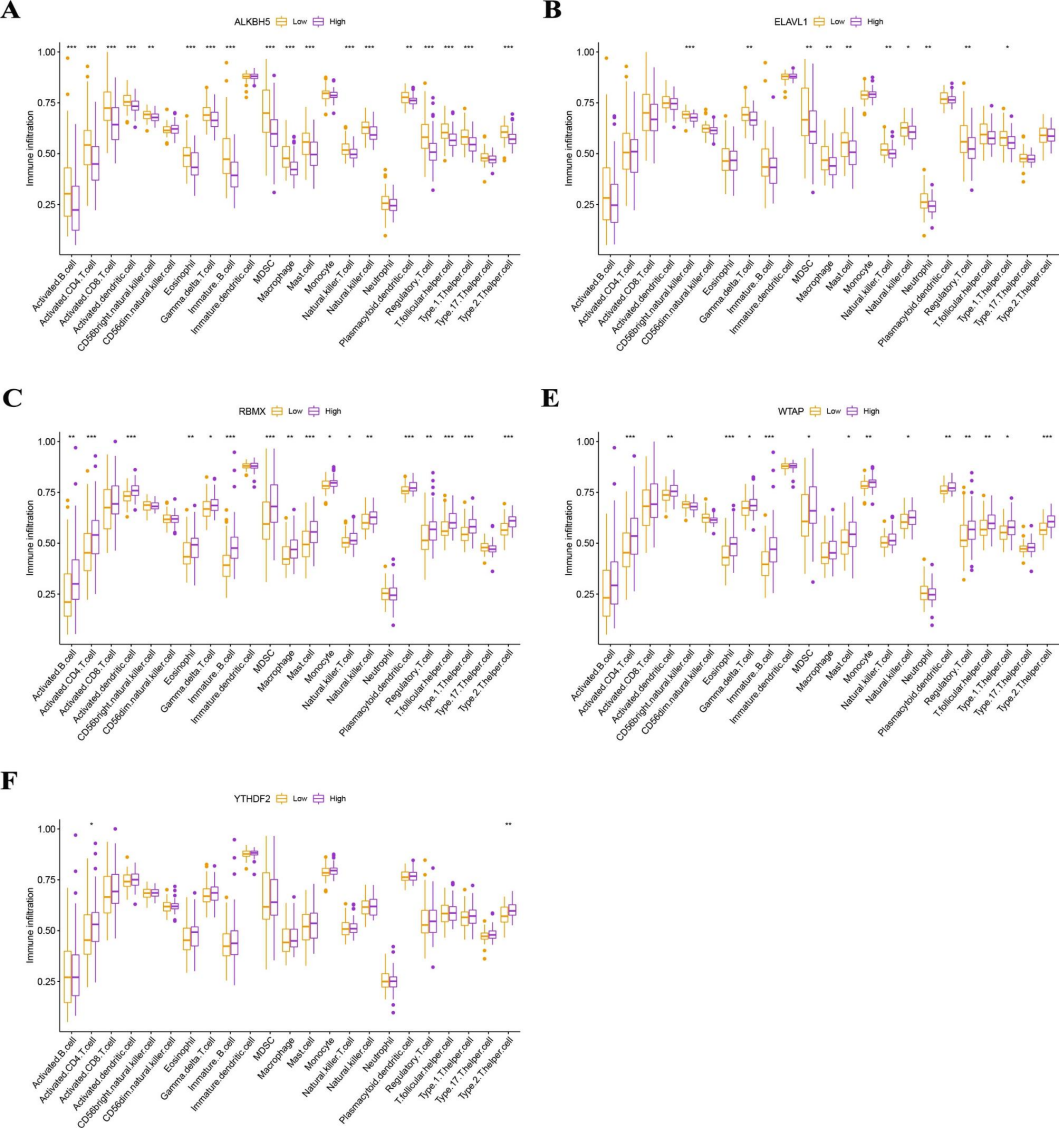
Correlation of the expression levels of five key MRGs with immune cell infiltration

Thereafter, we further evaluated their correlation with immune cell infiltration (Fig. 5).

### Function and pathway enrichment

**Fig. 6:**
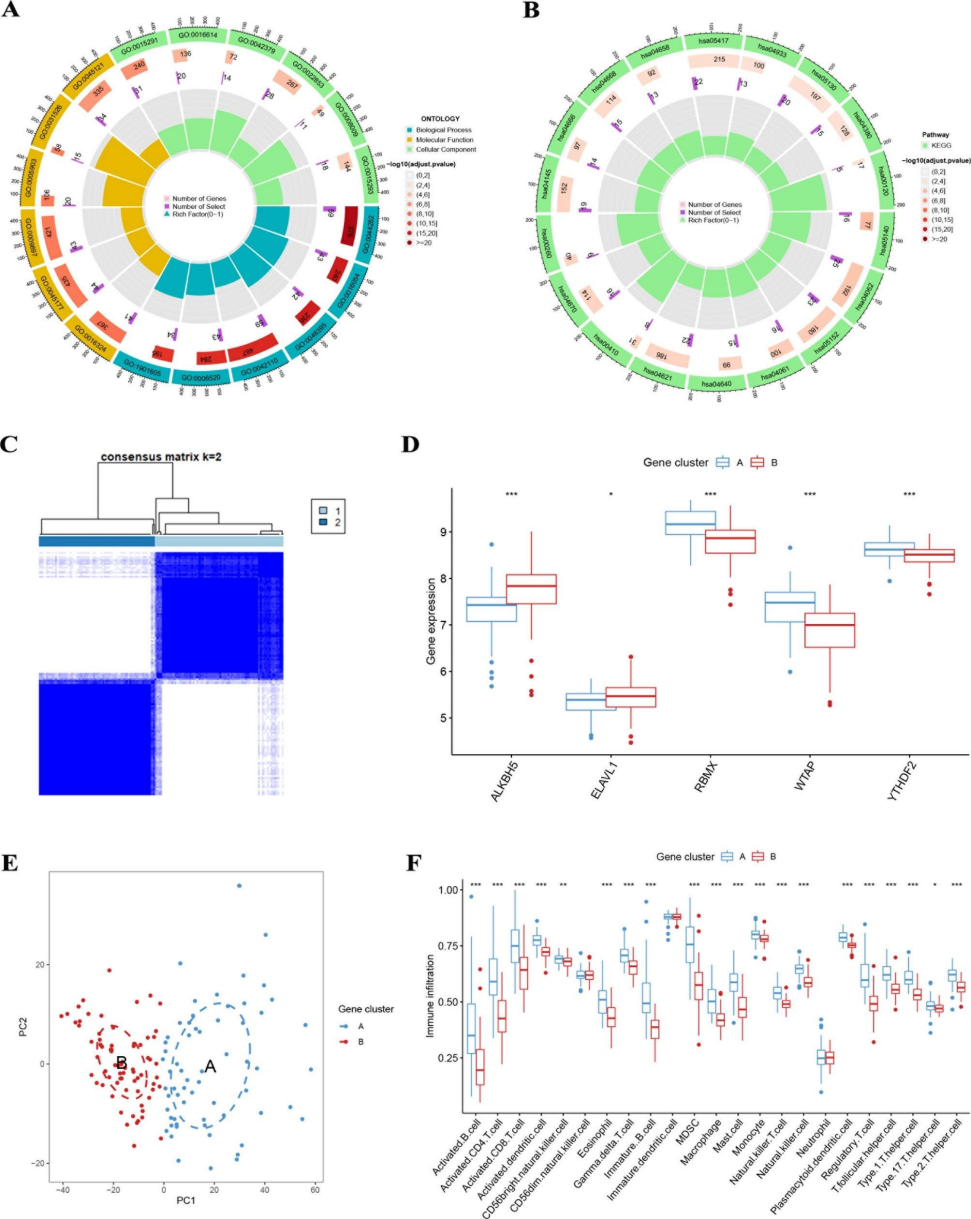
Consensus clustering of DEGs. (**A** and **B**) The GO and KEGG analyses for DEGs. (**C**) Consensus matrices of DEGs for k = 2. (**D**) A histogram of differential expression of the five key MRGs in gene clusters A and B. (**E**) Differences in immune cell infiltration between gene clusters A and B

A total of 768 DEGs were selected in two MRG patterns with thresholds of | logFC | greater than or equal to 0.585 and an adjusted p value greater than 0.05. We used GO and KEGG enrichment analyses to further investigate the probable roles and molecular pathways of these DEGs in RIF. Biological process (BP) terminology is associated with the small molecule catabolic process (GO: 0044282) and the organic acid catabolic process (GO: 0016054); cellular component (CC) terminology is associated with apical plasma membrane (GO: 0016324) and apical part of cell (GO: 0045177); molecular function (MF) terms are related to secondary active transmembrane transporter activity (GO: 0015291) and oxidoreductase activity, acting on CH-OH group of donors (GO: 0016614)(Fig. 6A **and Supplementary Table 3**). KEGG enrichment analysis revealed that DEGs were highly enriched in the leishmaniasis (hsa05140) and chemokine signaling (hsa04062) pathways (Fig. 6B **and Supplementary Table 4**).

### Identification of two distinct genetic patterns and immune cell infiltration

**Fig. 7:**
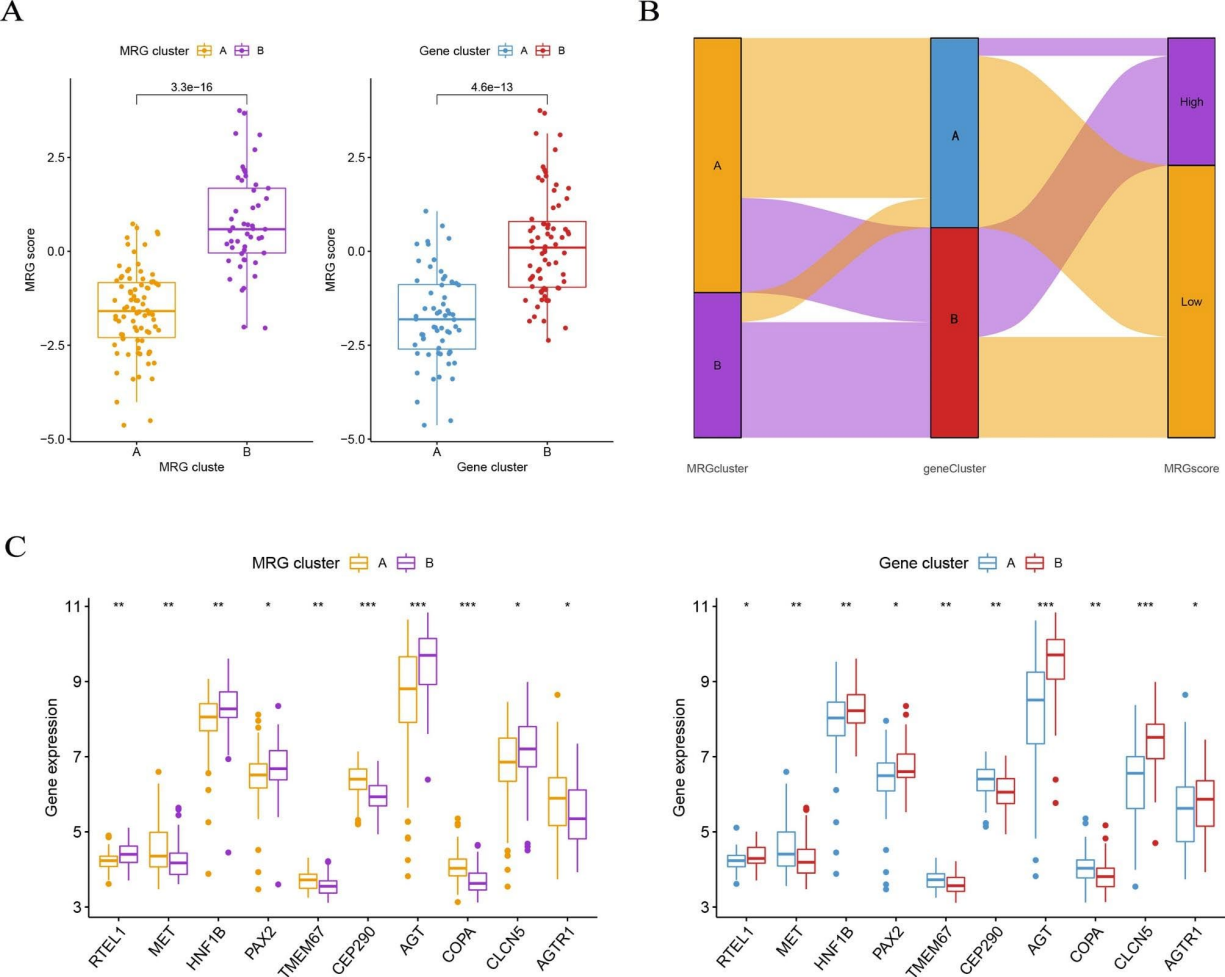
Role of MRG patterns in the identification of RIF. (**A**) There were differences in MRG scores between MRG gene clusters A and B and between gene clusters A and B. (**B**) A Sankey diagram showing the relationship between MRG patterns, gene patterns, and MRG scores. (**C**) Differences in expression levels of RTEL1, MET, HNF1B, PAX2, TMEM67, CEP290, AGT, COPA, CLCN5, and AGTR1 between MRG clusters A and B and between gene clusters A and B

To further verify the MRG pattern, patients with RIF were divided into different genomic subtypes based on 768 DEGs using a consistent clustering approach. We got two different gene patterns, gene cluster A and gene cluster B, which corresponded to the grouping of MRG patterns (Fig. 6C), while PCA revealed that the two gene patterns were fully distinct (Fig. 6E). Figure 6D F showed that the differential expression levels of the five key MRGs between gene cluster A and gene cluster B and the immune cell infiltration between the two gene patterns are similar to those in the MRG clusters. This again validates the correctness of the consensus clustering approach to grouping. To compute the MRG cluster, we used a PCA algorithm to compare the MRG scores between two different MRG clusters or gene clusters. The results showed that MRG cluster B, or gene cluster B, had a significantly higher MRG score than MRG cluster A, or gene cluster A (Fig. 7A). The relationship between MRG pattern, gene pattern, and MRG score is visualized in the Sankey plot (Fig. 7B).

### The role of MRG and gene patterns in RIF

To further investigate the connection between the MRG cluster and RIF, we explored the relationship between different clusters and the expression levels of genes closely related to the development of RIF, including RTEL1, MET, HNF1B, PAX2, TMEM67, CEP290, AGT, COPA, CLCN5, and AGTR1. The results showed that the expression levels of MET, TMEM67, CEP290, and COPA were higher in gene cluster A or MRG cluster A, while the rest were reversed, suggesting that gene cluster A or MRG cluster A is highly associated with RIF characteristics (Fig. 7 C **and D**).

## Discussion

RIF is a common pathological manifestation of CKD progressing to end-stage renal disease. Its pathogenesis is mainly related to renal inflammatory injury, oxidative stress, and apoptosis, but the most important pathogenesis is the imbalance of extracellular matrix synthesis and/or degradation, and excessive deposition in normal interstitium and tubules [1, 42, 43]. RNA methylation has emerged as a crucial molecular mechanism involved in diverse biological pathways, including regulation of stem cell homeostasis, cell differentiation, DNA damage response, and gene expression. Moreover, dysregulation of RNA methylation has been implicated in the pathogenesis of multiple diseases, such as liver fibrosis, colorectal cancer, gastric cancer, bladder cancer, and several others [44–46]. However, no study has yet identified the role and mechanism of m^1^A, m^6^A and m^5^C modifications in RIF.

We first identified 33m^1^A/m^6^A/m^5^C regulators by differential expression analysis between patients with and without RIF. Then we finally obtained 5 key MRGs (ALKBH5, ELAVL1, RBMX, WTAP, and YTHDF2) by applying RF, LASSO, SVM, and PPI networks. Subsequently, a nomogram model was constructed utilizing 5 MRGs, enabling the prediction of RIF onset. The DCA curves suggest that decision-making based on the nomogram model could prove advantageous for patients experiencing RIF. Furthermore, two distinct MRG patterns were identified based on the aforementioned 5 MRGs. The correlation between immune cell infiltration, and these two patterns, and the five MRGs was subsequently explored. In between the two MRG patterns, we screened an additional 768 DEGs and distinguished two different gene patterns using consensus clustering, and interestingly, this gene pattern was grouped in line with the MRG pattern.

WTAP is a widespread nuclear protein that localizes throughout the nucleoplasm as well as in patches and binds specifically to the Wilm’s tumor 1 protein [47]. As an essential component of the methyltransferase complex, it can interact directly with METTL3 to recruit METTL3-METTL14 heterodimeric complexes to nuclear speckles for m6A modification [8]. Moreover, WTAP is involved in selective splicing of mRNA and cell cycle regulation [48]. Wei et al. found that AcSDKP could reduce the stability of Ptch1 mRNA by downregulating WTAP expression and ultimately exert anti-fibrotic effects [49]. A gene on the X chromosome codes for RBMX, a nuclear RNA-binding protein with a length of 43 kDa. It supports genomic integrity, transcription regulation, and splicing at the molecular level and is directly linked to healthy development, cancer, and viral infections [50]. Renieri et al. used genome sequencing to find mutations truncating the RBMX gene in lung cancer patients [51]. According to Martinez-Arribas et al., pro-apoptotic Bax gene expression and RBMX expression were associated with breast cancer [52]. ALKBH5 belongs to the AlkB subfamily of the 2OG dioxygenase superfamily, also known as erasers, and contains a highly conserved DSBH fold (also known as the jelly-roll motif) in its structure, which maintains the intracellular homeostasis of m^6^A modifications with the assistance of Fe2 + and the cofactor 2OG in conjunction with the methyltransferase complex [53]. ALKBH5 can affect the development of human malignant diseases by regulating biological processes [54, 55]. ALKBH5 can contribute to the progression of lung fibrosis by directly or indirectly regulating FOXM1 [56]. Inhibition of ALKBH5 in hepatic stellate cells attenuates radiation-induced liver fibrosis [57].

HuR, the gene product of the ELAVL1 gene, is the only member of the entire ELAVL family that is commonly expressed as RBPs in all human tissues [58]. HuR contains three RRMs, which are translocated from the nucleus to the cytoplasm when exposed to intrinsic and/or extrinsic stress and bind to A/U-rich elements in the 3’ untranslated region of mRNA to regulate mRNA splicing, transport, and stability [59, 60]. A prior investigation on liver fibrosis revealed that HuR binds to the 3’UTR of S1PR3 mRNA, thereby enhancing its stability through competitive inhibition of miR-30e. This resultant regulation of signaling pathways mediated via S1P-S1PR3 subsequently impacts the migration and differentiation of BMSCs into myofibroblasts [61]. Sorafenib attenuates hepatic fibrosis in mice by inducing hepatic stellate cell ferroptosis [62]. IAPF overexpression can block autophagy in lung fibrosis, and the action is dependent on ELAVL1 [63]. This all suggests a potential role for ELAVL1 and HuR in the development of liver fibrosis. As the most efficient m^6^A “reader”, YTHDF2 can target m^6^A-containing RNAs through its C-terminal YTH structural domain to regulate RNA processing, stabilization, and translation [64, 65]. YTHDF2 functions in various biological processes, including cancer development, inflammatory responses, regulation of hematopoietic stem cell self-renewal and differentiation, and initiation of pluripotent stem cell generation [64].In conclusion, the above five m^1^A/m^6^A/m^5^C regulators had direct or indirect relationships with fibrosis.

The pathogenesis of interstitial fibrosis is strongly connected to abnormalities in the immune system, particularly those related to the regulatory mechanisms of mast cells. A significant aspect of this process involves the synthesis, storage, and release of reactive renin by mast cells, which cleaves angiotensinogen and contributes to the production of ANG II, a key pro-fibrotic factor associated with renal fibrosis. Recent research conducted by Veerappan et al. demonstrated that treatment with sodium cromoglycate, a mast cell stabilizer, resulted in a reduction of collagen deposition and tubulointerstitial fibrosis in obstructed kidneys after a 14-day period [66]. This finding highlights the critical role of mast cells in the development of interstitial fibrosis. According to a study by Hirooka et al., Foxp3+-Treg has a protective function in the pathophysiology of RIF by activating the L-18R signaling pathway [67]. Wang et al. found by bone marrow transplantation experiments that bone marrow-derived macrophages, especially M2-type macrophages, could transdifferentiate into renal myofibroblasts and promote RIF [68]. TGF-β1 is an essential mediator in the pathogenesis of RIF, with anti-inflammatory and pro-fibrotic effects [69]. In fact, TGF-β1 is associated with a variety of immune cells, including B cells, CD8 + T cells, regulatory CD4 + T cells, and dendritic cells. For example, TGF-β1 can induce cell cycle arrest in B cells by inhibiting PI3K/Akt signaling, especially in the G0/G1 phase [70]. In addition, TGF-β1 can also indirectly impede B cell proliferation and activation by interacting with regulatory T cells [71]. In conclusion, given the importance of TGF-f1 in the immune cascade response, we know that the pathogenesis of RIF is associated with multiple immune cell infiltrations.

In our study, two MRG patterns were recognized by using a consensus clustering approach on five selected key MRGs. The high level of multiple immune cell infiltration in MRG cluster A suggests that MRG cluster A may be associated with RIF. The accuracy of the aforementioned results was then verified using DEGs connected to the gene pattern. By utilizing the PCA algorithm to determine the MRG score for each sample, we finally quantified the MRG pattern. Finally, we discovered that MRG cluster or gene cluster A had lower MRG scores. However, there are still some shortcomings in the study. First, since the datasets used do not have clinical data, including age, gender, glomerular filtration rate, etc., there is no way to further investigate the relationship between this clinical information and the different subtypes. Second, the sample size of this study is small, which may lead to some bias. Third, the results of the study still need to be validated by clinical cohort studies and in vitro and in vivo experiments. Therefore, we will collect more clinical samples, record more clinical information, and conduct in vitro and in vivo experiments and clinical cohort studies to further test our results.

## Conclusion

To sum up, our investigation successfully identified five significant MRGs and developed a nomogram model to predict the incidence of RIF in patients. Additionally, two distinct MRG patterns were identified based on the aforementioned five MRGs, with MRG cluster A potentially linked to RIF. Collectively, our findings suggest that MRGs could serve as a viable diagnostic tool for identifying RIF subtypes, thus contributing to its prevention, delay, and treatment.

## Electronic supplementary material

Below is the link to the electronic supplementary material.

Additional file 1: Supplementary Figure 1. (A-G) Consensus matrices of the 5 MRGs for k = 3-9. Supplementary Figure 2. (A-G) Consensus matrices of the 56 DEGs for k = 3-9.

Additional file 2:Supplementary Table 1. The 48 MRGs. Supplementary Table 2. The 33 differentially expressed MRGs. Supplementary Table 3. The GO enrichment analysis. Supplementary Table 4. The KEGG enrichment analysis.

Supplementary Material 3

## Data Availability

The raw data used and analyzed during the current study are available from the corresponding author on reasonable request.
