# Supplementary figures and images for: Significance of methylation-related genes in diagnosis and subtype classification of renal interstitial fibrosis
Source: Hereditas. 2023 Jul 27;160:32. doi: 10.1186/s41065-023-00295-8 (PMC10373342; doi:10.1186/s41065-023-00295-8)

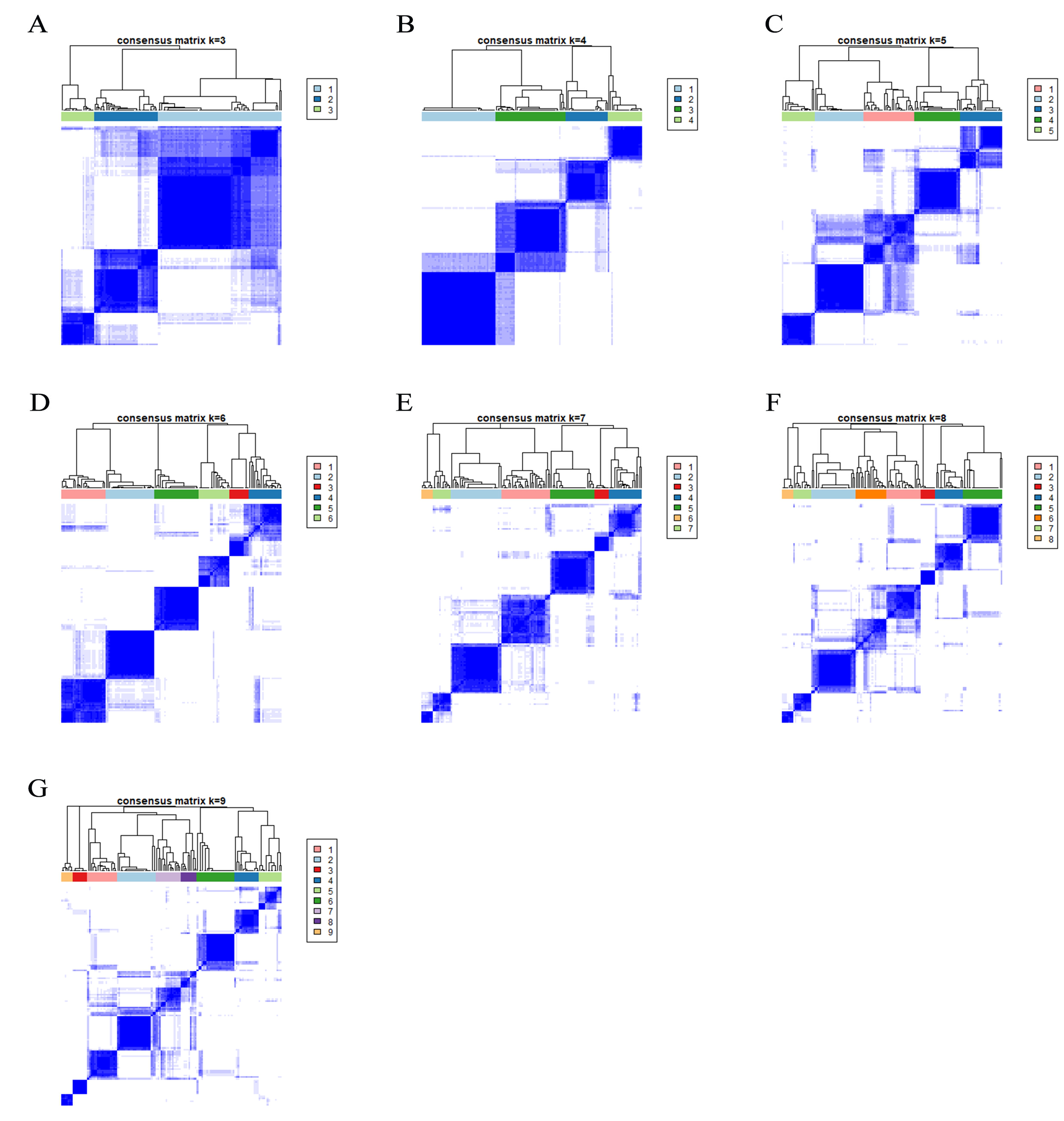

Supplement: Supplementary file 1 — Additional file 1: Supplementary Figure 1. (A-G) Consensus matrices of the 5 MRGs for k = 3-9. Supplementary Figure 2. (A-G) Consensus matrices of the 56 DEGs for k = 3-9. [file 41065_2023_295_MOESM1_ESM.png]

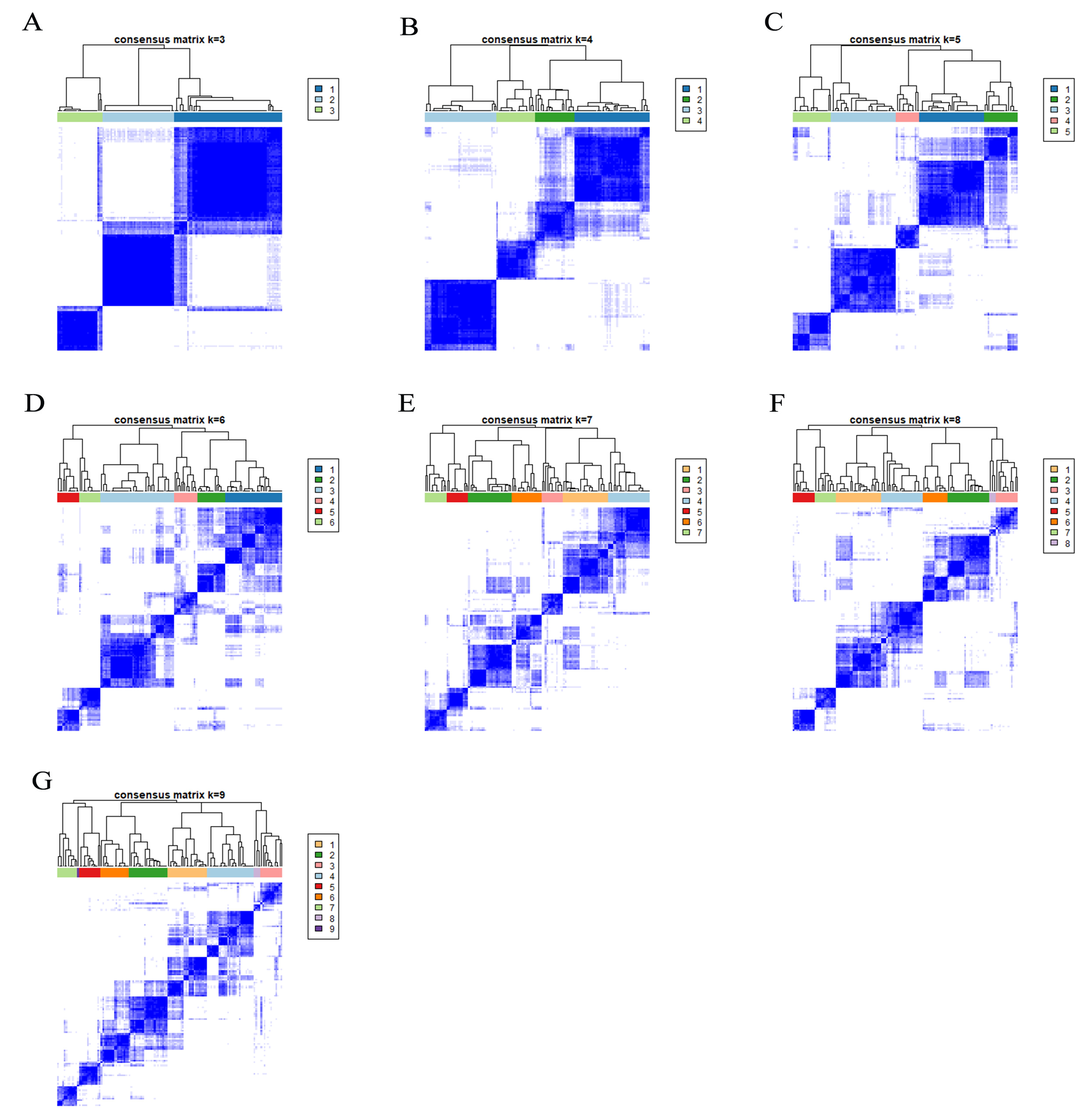

Supplement: Supplementary file 2 — Additional file 2:Supplementary Table 1. The 48 MRGs. Supplementary Table 2. The 33 differentially expressed MRGs. Supplementary Table 3. The GO enrichment analysis. Supplementary Table 4. The KEGG enrichment analysis. [file 41065_2023_295_MOESM2_ESM.png]
