# Supplementary material for: Significance of methylation-related genes in diagnosis and subtype classification of renal interstitial fibrosis
Source: Hereditas. 2023 Jul 27;160:32. doi: 10.1186/s41065-023-00295-8 (PMC10373342; doi:10.1186/s41065-023-00295-8)
Supplement: Supplementary file 3 — Supplementary Material 3 [file 41065_2023_295_MOESM3_ESM.docx]

Supplementary Figure 1. Consensus clustering of the 5 MRGs. (A-G) Consensus matrices of the 5 MRGs for k = 3-9.





Supplementary Figure 2. Consensus clustering of the 768 DEGs. (A-G) Consensus matrices of the 56 DEGs for k = 3-9.

| Gene Symbol | Gene Name | Type |
| --- | --- | --- |
| METTL3 | Methyltransferase 3, N6-Adenosine-Methyltransferase Complex Catalytic Subunit | m^1^A/m^6^A/m^5^C |
| METTL14 | Methyltransferase 14, N6-Adenosine-Methyltransferase Subunit | m^1^A/m^6^A/m^5^C |
| WTAP | WT1 Associated Protein | m^1^A/m^6^A/m^5^C |
| KIAA1429 | Vir Like M6A Methyltransferase Associated | m^1^A/m^6^A/m^5^C |
| RBM15 | RNA Binding Motif Protein 15 | m^1^A/m^6^A/m^5^C |
| RBM15B | RNA Binding Motif Protein 15B | m^1^A/m^6^A/m^5^C |
| ZC3H13 | Zinc Finger CCCH-Type Containing 13 | m^1^A/m^6^A/m^5^C |
| FTO | FTO Alpha-Ketoglutarate Dependent Dioxygenase | m^1^A/m^6^A/m^5^C |
| ALKBH5 | AlkB Homolog 5, RNA Demethylase | m^1^A/m^6^A/m^5^C |
| YTHDC1 | YTH N6-Methyladenosine RNA Binding Protein C1 | m^1^A/m^6^A/m^5^C |
| YTHDC2 | YTH N6-Methyladenosine RNA Binding Protein C2 | m^1^A/m^6^A/m^5^C |
| YTHDF1 | YTH N6-Methyladenosine RNA Binding Protein F1 | m^1^A/m^6^A/m^5^C |
| YTHDF2 | YTH N6-Methyladenosine RNA Binding Protein F2 | m^1^A/m^6^A/m^5^C |
| YTHDF3 | YTH N6-Methyladenosine RNA Binding Protein F3 | m^1^A/m^6^A/m^5^C |
| IGF2BP1 | Insulin Like Growth Factor 2 MRNA Binding Protein 1 | m^1^A/m^6^A/m^5^C |
| IGF2BP2 | Insulin Like Growth Factor 2 MRNA Binding Protein 2 | m^1^A/m^6^A/m^5^C |
| IGF2BP3 | Insulin Like Growth Factor 2 MRNA Binding Protein 3 | m^1^A/m^6^A/m^5^C |
| HNRNPA2B1 | Heterogeneous Nuclear Ribonucleoprotein A2/B1 | m^1^A/m^6^A/m^5^C |
| HNRNPC | Heterogeneous Nuclear Ribonucleoprotein C | m^1^A/m^6^A/m^5^C |
| RBMX | RNA Binding Motif Protein X-Linked | m^1^A/m^6^A/m^5^C |
| LRPPRC | Leucine Rich Pentatricopeptide Repeat Containing | m^1^A/m^6^A/m^5^C |
| FMR1 | Fragile X Messenger Ribonucleoprotein 1 | m^1^A/m^6^A/m^5^C |
| CBLL1 | Cbl Proto-Oncogene Like 1 | m^1^A/m^6^A/m^5^C |
| IGFBP1 | Insulin Like Growth Factor Binding Protein 1 | m^1^A/m^6^A/m^5^C |
| IGFBP2 | Insulin Like Growth Factor Binding Protein 2 | m^1^A/m^6^A/m^5^C |
| IGFBP3 | Insulin Like Growth Factor Binding Protein 3 | m^1^A/m^6^A/m^5^C |
| ELAVL1 | ELAV Like RNA Binding Protein 1 | m^1^A/m^6^A/m^5^C |
| TRMT6 | TRNA Methyltransferase 6 Non-Catalytic Subunit | m^1^A/m^6^A/m^5^C |
| TRMT61A | TRNA Methyltransferase 61A | m^1^A/m^6^A/m^5^C |
| TRMT61B | TRNA Methyltransferase 61B | m^1^A/m^6^A/m^5^C |
| RRP8 | Ribosomal RNA Processing 8 | m^1^A/m^6^A/m^5^C |
| ALKBH1 | AlkB Homolog 1, Histone H2A Dioxygenase | m^1^A/m^6^A/m^5^C |
| ALKBH3 | AlkB Homolog 3, Alpha-Ketoglutarate Dependent Dioxygenase | m^1^A/m^6^A/m^5^C |
| NOP2 | NOP2 Nucleolar Protein | m^1^A/m^6^A/m^5^C |
| NSUN2 | NOP2/Sun RNA Methyltransferase 2 | m^1^A/m^6^A/m^5^C |
| NSUN3 | NOP2/Sun RNA Methyltransferase 3 | m^1^A/m^6^A/m^5^C |
| NSUN4 | NOP2/Sun RNA Methyltransferase 4 | m^1^A/m^6^A/m^5^C |
| NSUN5 | NOP2/Sun RNA Methyltransferase 5 | m^1^A/m^6^A/m^5^C |
| NSUN7 | NOP2/Sun RNA Methyltransferase 7 | m^1^A/m^6^A/m^5^C |
| DNMT1 | DNA Methyltransferase 1 | m^1^A/m^6^A/m^5^C |
| TRDMT1 | TRNA Aspartic Acid Methyltransferase 1 | m^1^A/m^6^A/m^5^C |
| DNMT3A | DNA Methyltransferase 3 Alpha | m^1^A/m^6^A/m^5^C |
| DNMT3B | DNA Methyltransferase 3 Beta | m^1^A/m^6^A/m^5^C |
| TET2 | Tet Methylcytosine Dioxygenase 2 | m^1^A/m^6^A/m^5^C |
| YBX1 | Y-Box Binding Protein 1 | m^1^A/m^6^A/m^5^C |
| NSUN6 | NOP2/Sun RNA Methyltransferase 6 | m^1^A/m^6^A/m^5^C |
| TET1 | Tet Methylcytosine Dioxygenase 1 | m^1^A/m^6^A/m^5^C |
| TET3 | Tet Methylcytosine Dioxygenase 3 | m^1^A/m^6^A/m^5^C |

Supplementary Table 1. The list of 48 MRGs.

| gene | pValue |
| --- | --- |
| DNMT1 | 6.74E-15 |
| ALKBH5 | 1.73E-11 |
| ELAVL1 | 2.76E-11 |
| LRPPRC | 3.56E-11 |
| IGF2BP3 | 3.05E-08 |
| NSUN5 | 5.63E-08 |
| NSUN7 | 5.76E-07 |
| METTL3 | 4.30E-06 |
| DNMT3A | 1.38E-05 |
| RBMX | 2.24E-05 |
| NOP2 | 2.66E-05 |
| TRMT61B | 2.67E-05 |
| YTHDF2 | 2.84E-05 |
| IGF2BP1 | 3.43E-05 |
| TRMT61A | 6.07E-05 |
| HNRNPA2B1 | 8.85E-05 |
| YTHDF3 | 0.000131661 |
| RRP8 | 0.000271003 |
| TET2 | 0.00034751 |
| HNRNPC | 0.000401736 |
| NSUN4 | 0.001024019 |
| WTAP | 0.001622109 |
| RBM15B | 0.00413726 |
| ALKBH1 | 0.004413677 |
| NSUN3 | 0.004822907 |
| IGFBP3 | 0.005346161 |
| TRMT6 | 0.012180791 |
| IGF2BP2 | 0.01495106 |
| FMR1 | 0.017645413 |
| KIAA1429 | 0.019246738 |
| RBM15 | 0.019449289 |
| YTHDF1 | 0.039190358 |
| YTHDC1 | 0.04880364 |

Supplementary Table 2. The details of 33 differentially expressed MRGs.

| ONTOLOGY | ID | Description | p.adjust | Count |
| --- | --- | --- | --- | --- |
| BP | GO:0044282 | small molecule catabolic process | 1.41E-18 | 59 |
| BP | GO:0016054 | organic acid catabolic process | 2.39E-15 | 43 |
| BP | GO:0046395 | carboxylic acid catabolic process | 5.26E-15 | 42 |
| BP | GO:0042110 | T cell activation | 1.24E-13 | 59 |
| BP | GO:0006520 | cellular amino acid metabolic process | 5.95E-13 | 43 |
| BP | GO:1901605 | alpha-amino acid metabolic process | 8.39E-12 | 34 |
| BP | GO:0019221 | cytokine-mediated signaling pathway | 6.37E-11 | 53 |
| BP | GO:0002274 | myeloid leukocyte activation | 3.56E-10 | 34 |
| BP | GO:0009063 | cellular amino acid catabolic process | 2.41E-09 | 23 |
| BP | GO:1901606 | alpha-amino acid catabolic process | 8.59E-09 | 20 |
| BP | GO:0071346 | cellular response to interferon-gamma | 9.10E-09 | 23 |
| BP | GO:0034341 | response to interferon-gamma | 9.92E-09 | 25 |
| BP | GO:0007159 | leukocyte cell-cell adhesion | 1.07E-08 | 42 |
| BP | GO:0050863 | regulation of T cell activation | 1.27E-08 | 39 |
| BP | GO:0045088 | regulation of innate immune response | 1.27E-08 | 31 |
| BP | GO:1903131 | mononuclear cell differentiation | 1.74E-08 | 45 |
| BP | GO:1903037 | regulation of leukocyte cell-cell adhesion | 1.99E-08 | 39 |
| BP | GO:0002831 | regulation of response to biotic stimulus | 3.20E-08 | 38 |
| BP | GO:0022409 | positive regulation of cell-cell adhesion | 3.26E-08 | 35 |
| BP | GO:0030098 | lymphocyte differentiation | 3.26E-08 | 41 |
| BP | GO:1903039 | positive regulation of leukocyte cell-cell adhesion | 9.60E-08 | 31 |
| BP | GO:0009615 | response to virus | 2.03E-07 | 39 |
| BP | GO:0022407 | regulation of cell-cell adhesion | 2.03E-07 | 44 |
| BP | GO:0052548 | regulation of endopeptidase activity | 2.03E-07 | 43 |
| BP | GO:0002699 | positive regulation of immune effector process | 2.27E-07 | 30 |
| BP | GO:0002764 | immune response-regulating signaling pathway | 2.28E-07 | 45 |
| BP | GO:0050867 | positive regulation of cell activation | 2.46E-07 | 42 |
| BP | GO:0006631 | fatty acid metabolic process | 2.83E-07 | 40 |
| BP | GO:0002443 | leukocyte mediated immunity | 2.94E-07 | 43 |
| BP | GO:0030217 | T cell differentiation | 4.06E-07 | 31 |
| BP | GO:0070661 | leukocyte proliferation | 4.34E-07 | 35 |
| BP | GO:0046651 | lymphocyte proliferation | 4.35E-07 | 33 |
| BP | GO:0050870 | positive regulation of T cell activation | 4.36E-07 | 28 |
| BP | GO:0001819 | positive regulation of cytokine production | 4.93E-07 | 44 |
| BP | GO:0002683 | negative regulation of immune system process | 4.93E-07 | 42 |
| BP | GO:0032943 | mononuclear cell proliferation | 4.93E-07 | 33 |
| BP | GO:0050900 | leukocyte migration | 4.93E-07 | 38 |
| BP | GO:0002697 | regulation of immune effector process | 5.50E-07 | 36 |
| BP | GO:2000116 | regulation of cysteine-type endopeptidase activity | 6.06E-07 | 29 |
| BP | GO:0034612 | response to tumor necrosis factor | 8.25E-07 | 30 |
| BP | GO:0052547 | regulation of peptidase activity | 8.49E-07 | 43 |
| BP | GO:0002768 | immune response-regulating cell surface receptor signaling pathway | 8.90E-07 | 34 |
| BP | GO:0002456 | T cell mediated immunity | 9.05E-07 | 19 |
| BP | GO:0002703 | regulation of leukocyte mediated immunity | 9.05E-07 | 28 |
| BP | GO:0006575 | cellular modified amino acid metabolic process | 1.33E-06 | 25 |
| BP | GO:0070663 | regulation of leukocyte proliferation | 1.33E-06 | 29 |
| BP | GO:0009636 | response to toxic substance | 1.58E-06 | 30 |
| BP | GO:0002253 | activation of immune response | 1.92E-06 | 37 |
| BP | GO:0002696 | positive regulation of leukocyte activation | 1.98E-06 | 39 |
| BP | GO:0046394 | carboxylic acid biosynthetic process | 2.39E-06 | 33 |
| BP | GO:0043281 | regulation of cysteine-type endopeptidase activity involved in apoptotic process | 2.44E-06 | 26 |
| BP | GO:0016053 | organic acid biosynthetic process | 2.68E-06 | 33 |
| BP | GO:0050670 | regulation of lymphocyte proliferation | 2.74E-06 | 27 |
| BP | GO:0032944 | regulation of mononuclear cell proliferation | 3.25E-06 | 27 |
| BP | GO:0030595 | leukocyte chemotaxis | 4.21E-06 | 27 |
| BP | GO:0006909 | phagocytosis | 4.56E-06 | 32 |
| BP | GO:0072329 | monocarboxylic acid catabolic process | 4.61E-06 | 19 |
| BP | GO:0050766 | positive regulation of phagocytosis | 4.81E-06 | 14 |
| BP | GO:0031341 | regulation of cell killing | 5.12E-06 | 17 |
| BP | GO:0009064 | glutamine family amino acid metabolic process | 6.30E-06 | 15 |
| BP | GO:0002237 | response to molecule of bacterial origin | 6.49E-06 | 35 |
| BP | GO:1904407 | positive regulation of nitric oxide metabolic process | 9.07E-06 | 11 |
| BP | GO:0045785 | positive regulation of cell adhesion | 9.07E-06 | 39 |
| BP | GO:0046209 | nitric oxide metabolic process | 9.99E-06 | 15 |
| BP | GO:0001910 | regulation of leukocyte mediated cytotoxicity | 1.14E-05 | 15 |
| BP | GO:2001057 | reactive nitrogen species metabolic process | 1.14E-05 | 15 |
| BP | GO:0002366 | leukocyte activation involved in immune response | 1.14E-05 | 29 |
| BP | GO:0002429 | immune response-activating cell surface receptor signaling pathway | 1.14E-05 | 30 |
| BP | GO:0002757 | immune response-activating signal transduction | 1.14E-05 | 30 |
| BP | GO:0042116 | macrophage activation | 1.21E-05 | 17 |
| BP | GO:0006525 | arginine metabolic process | 1.34E-05 | 8 |
| BP | GO:0050764 | regulation of phagocytosis | 1.34E-05 | 16 |
| BP | GO:0032496 | response to lipopolysaccharide | 1.34E-05 | 33 |
| BP | GO:0060333 | interferon-gamma-mediated signaling pathway | 1.39E-05 | 9 |
| BP | GO:0002263 | cell activation involved in immune response | 1.42E-05 | 29 |
| BP | GO:0006691 | leukotriene metabolic process | 1.47E-05 | 10 |
| BP | GO:0031343 | positive regulation of cell killing | 1.50E-05 | 13 |
| BP | GO:0050854 | regulation of antigen receptor-mediated signaling pathway | 1.50E-05 | 13 |
| BP | GO:0042554 | superoxide anion generation | 1.58E-05 | 11 |
| BP | GO:1990266 | neutrophil migration | 1.70E-05 | 18 |
| BP | GO:0048247 | lymphocyte chemotaxis | 1.73E-05 | 13 |
| BP | GO:0080164 | regulation of nitric oxide metabolic process | 1.73E-05 | 13 |
| BP | GO:0006968 | cellular defense response | 1.74E-05 | 12 |
| BP | GO:0001909 | leukocyte mediated cytotoxicity | 2.08E-05 | 18 |
| BP | GO:0070098 | chemokine-mediated signaling pathway | 2.33E-05 | 15 |
| BP | GO:0001912 | positive regulation of leukocyte mediated cytotoxicity | 2.56E-05 | 12 |
| BP | GO:0002819 | regulation of adaptive immune response | 3.06E-05 | 22 |
| BP | GO:0071356 | cellular response to tumor necrosis factor | 3.30E-05 | 25 |
| BP | GO:0030593 | neutrophil chemotaxis | 3.41E-05 | 16 |
| BP | GO:0072503 | cellular divalent inorganic cation homeostasis | 3.62E-05 | 40 |
| BP | GO:0051346 | negative regulation of hydrolase activity | 3.62E-05 | 34 |
| BP | GO:1902105 | regulation of leukocyte differentiation | 3.82E-05 | 28 |
| BP | GO:0002695 | negative regulation of leukocyte activation | 4.15E-05 | 22 |
| BP | GO:0001913 | T cell mediated cytotoxicity | 4.28E-05 | 11 |
| BP | GO:0051607 | defense response to virus | 4.28E-05 | 27 |
| BP | GO:0140546 | defense response to symbiont | 4.28E-05 | 27 |
| BP | GO:0045429 | positive regulation of nitric oxide biosynthetic process | 4.53E-05 | 10 |
| BP | GO:0002449 | lymphocyte mediated immunity | 4.72E-05 | 32 |
| BP | GO:2001056 | positive regulation of cysteine-type endopeptidase activity | 5.80E-05 | 19 |
| BP | GO:0015849 | organic acid transport | 5.93E-05 | 29 |
| BP | GO:0032731 | positive regulation of interleukin-1 beta production | 6.80E-05 | 12 |
| BP | GO:0045428 | regulation of nitric oxide biosynthetic process | 6.80E-05 | 12 |
| BP | GO:0010950 | positive regulation of endopeptidase activity | 6.80E-05 | 21 |
| BP | GO:1990868 | response to chemokine | 6.80E-05 | 15 |
| BP | GO:1990869 | cellular response to chemokine | 6.80E-05 | 15 |
| BP | GO:0050777 | negative regulation of immune response | 6.80E-05 | 22 |
| BP | GO:0002709 | regulation of T cell mediated immunity | 6.89E-05 | 14 |
| BP | GO:0050866 | negative regulation of cell activation | 7.12E-05 | 23 |
| BP | GO:0006801 | superoxide metabolic process | 7.51E-05 | 13 |
| BP | GO:0051251 | positive regulation of lymphocyte activation | 8.72E-05 | 32 |
| BP | GO:0042098 | T cell proliferation | 9.95E-05 | 22 |
| BP | GO:0006809 | nitric oxide biosynthetic process | 9.99E-05 | 13 |
| BP | GO:0055074 | calcium ion homeostasis | 1.30E-04 | 37 |
| BP | GO:1901616 | organic hydroxy compound catabolic process | 1.33E-04 | 13 |
| BP | GO:0090322 | regulation of superoxide metabolic process | 1.35E-04 | 9 |
| BP | GO:0001906 | cell killing | 1.36E-04 | 21 |
| BP | GO:0032103 | positive regulation of response to external stimulus | 1.53E-04 | 35 |
| BP | GO:0071219 | cellular response to molecule of bacterial origin | 1.56E-04 | 23 |
| BP | GO:0002460 | adaptive immune response based on somatic recombination of immune receptors built from immunoglobulin superfamily domains | 1.59E-04 | 31 |
| BP | GO:0045580 | regulation of T cell differentiation | 1.63E-04 | 18 |
| BP | GO:0006874 | cellular calcium ion homeostasis | 1.69E-04 | 36 |
| BP | GO:0001774 | microglial cell activation | 1.76E-04 | 10 |
| BP | GO:0050851 | antigen receptor-mediated signaling pathway | 1.86E-04 | 24 |
| BP | GO:0097530 | granulocyte migration | 1.91E-04 | 18 |
| BP | GO:0005996 | monosaccharide metabolic process | 1.91E-04 | 25 |
| BP | GO:0002705 | positive regulation of leukocyte mediated immunity | 1.94E-04 | 17 |
| BP | GO:1905039 | carboxylic acid transmembrane transport | 2.06E-04 | 18 |
| BP | GO:0060326 | cell chemotaxis | 2.16E-04 | 28 |
| BP | GO:1903825 | organic acid transmembrane transport | 2.23E-04 | 18 |
| BP | GO:0001914 | regulation of T cell mediated cytotoxicity | 2.44E-04 | 9 |
| BP | GO:1901361 | organic cyclic compound catabolic process | 2.46E-04 | 38 |
| BP | GO:0002718 | regulation of cytokine production involved in immune response | 2.46E-04 | 14 |
| BP | GO:0010952 | positive regulation of peptidase activity | 2.48E-04 | 21 |
| BP | GO:0006690 | icosanoid metabolic process | 2.48E-04 | 16 |
| BP | GO:0071216 | cellular response to biotic stimulus | 2.58E-04 | 24 |
| BP | GO:1903706 | regulation of hemopoiesis | 2.58E-04 | 31 |
| BP | GO:0002706 | regulation of lymphocyte mediated immunity | 2.76E-04 | 19 |
| BP | GO:0002822 | regulation of adaptive immune response based on somatic recombination of immune receptors built from immunoglobulin superfamily domains | 2.76E-04 | 19 |
| BP | GO:0002833 | positive regulation of response to biotic stimulus | 2.76E-04 | 19 |
| BP | GO:0034113 | heterotypic cell-cell adhesion | 2.81E-04 | 11 |
| BP | GO:0071621 | granulocyte chemotaxis | 2.91E-04 | 16 |
| BP | GO:0002367 | cytokine production involved in immune response | 2.91E-04 | 14 |
| BP | GO:0032732 | positive regulation of interleukin-1 production | 2.91E-04 | 12 |
| BP | GO:0043299 | leukocyte degranulation | 2.91E-04 | 12 |
| BP | GO:0042100 | B cell proliferation | 3.22E-04 | 14 |
| BP | GO:0007204 | positive regulation of cytosolic calcium ion concentration | 3.25E-04 | 28 |
| BP | GO:0071634 | regulation of transforming growth factor beta production | 3.35E-04 | 9 |
| BP | GO:0042129 | regulation of T cell proliferation | 3.35E-04 | 19 |
| BP | GO:0046631 | alpha-beta T cell activation | 3.36E-04 | 18 |
| BP | GO:0035747 | natural killer cell chemotaxis | 3.74E-04 | 5 |
| BP | GO:0097529 | myeloid leukocyte migration | 3.74E-04 | 22 |
| BP | GO:0019318 | hexose metabolic process | 3.87E-04 | 23 |
| BP | GO:0043280 | positive regulation of cysteine-type endopeptidase activity involved in apoptotic process | 4.02E-04 | 16 |
| BP | GO:0045619 | regulation of lymphocyte differentiation | 4.14E-04 | 19 |
| BP | GO:0032928 | regulation of superoxide anion generation | 4.19E-04 | 7 |
| BP | GO:0046942 | carboxylic acid transport | 4.39E-04 | 25 |
| BP | GO:0032760 | positive regulation of tumor necrosis factor production | 4.68E-04 | 14 |
| BP | GO:0036230 | granulocyte activation | 4.68E-04 | 9 |
| BP | GO:0071604 | transforming growth factor beta production | 4.68E-04 | 9 |
| BP | GO:0045089 | positive regulation of innate immune response | 4.68E-04 | 16 |
| BP | GO:0072676 | lymphocyte migration | 4.75E-04 | 15 |
| BP | GO:0043270 | positive regulation of ion transport | 4.79E-04 | 25 |
| BP | GO:0071222 | cellular response to lipopolysaccharide | 5.06E-04 | 21 |
| BP | GO:0002275 | myeloid cell activation involved in immune response | 5.27E-04 | 13 |
| BP | GO:1901623 | regulation of lymphocyte chemotaxis | 5.30E-04 | 7 |
| BP | GO:0006816 | calcium ion transport | 5.38E-04 | 33 |
| BP | GO:0098656 | anion transmembrane transport | 6.03E-04 | 23 |
| BP | GO:0019882 | antigen processing and presentation | 6.17E-04 | 14 |
| BP | GO:0001959 | regulation of cytokine-mediated signaling pathway | 6.41E-04 | 17 |
| BP | GO:0070665 | positive regulation of leukocyte proliferation | 6.41E-04 | 17 |
| BP | GO:0002886 | regulation of myeloid leukocyte mediated immunity | 6.41E-04 | 10 |
| BP | GO:0046456 | icosanoid biosynthetic process | 6.41E-04 | 10 |
| BP | GO:0032640 | tumor necrosis factor production | 6.41E-04 | 19 |
| BP | GO:0032680 | regulation of tumor necrosis factor production | 6.41E-04 | 19 |
| BP | GO:0044273 | sulfur compound catabolic process | 6.41E-04 | 8 |
| BP | GO:0050730 | regulation of peptidyl-tyrosine phosphorylation | 6.45E-04 | 24 |
| BP | GO:0002824 | positive regulation of adaptive immune response based on somatic recombination of immune receptors built from immunoglobulin superfamily domains | 6.45E-04 | 14 |
| BP | GO:1903557 | positive regulation of tumor necrosis factor superfamily cytokine production | 6.45E-04 | 14 |
| BP | GO:0051480 | regulation of cytosolic calcium ion concentration | 6.45E-04 | 29 |
| BP | GO:0051651 | maintenance of location in cell | 6.55E-04 | 21 |
| BP | GO:0015711 | organic anion transport | 6.72E-04 | 29 |
| BP | GO:0050671 | positive regulation of lymphocyte proliferation | 7.23E-04 | 16 |
| BP | GO:0009612 | response to mechanical stimulus | 7.40E-04 | 21 |
| BP | GO:0016042 | lipid catabolic process | 7.46E-04 | 27 |
| BP | GO:0050852 | T cell receptor signaling pathway | 7.52E-04 | 15 |
| BP | GO:0042119 | neutrophil activation | 7.52E-04 | 8 |
| BP | GO:0046364 | monosaccharide biosynthetic process | 7.54E-04 | 12 |
| BP | GO:0032946 | positive regulation of mononuclear cell proliferation | 7.67E-04 | 16 |
| BP | GO:0010951 | negative regulation of endopeptidase activity | 8.27E-04 | 23 |
| BP | GO:0032611 | interleukin-1 beta production | 8.27E-04 | 14 |
| BP | GO:0032651 | regulation of interleukin-1 beta production | 8.27E-04 | 14 |
| BP | GO:0019884 | antigen processing and presentation of exogenous antigen | 8.27E-04 | 9 |
| BP | GO:0071706 | tumor necrosis factor superfamily cytokine production | 8.42E-04 | 19 |
| BP | GO:1903555 | regulation of tumor necrosis factor superfamily cytokine production | 8.42E-04 | 19 |
| BP | GO:0045730 | respiratory burst | 8.87E-04 | 8 |
| BP | GO:0045824 | negative regulation of innate immune response | 8.94E-04 | 11 |
| BP | GO:0002821 | positive regulation of adaptive immune response | 9.84E-04 | 14 |
| BP | GO:0009065 | glutamine family amino acid catabolic process | 9.92E-04 | 7 |
| BP | GO:0051250 | negative regulation of lymphocyte activation | 9.92E-04 | 17 |
| BP | GO:1902107 | positive regulation of leukocyte differentiation | 9.92E-04 | 17 |
| BP | GO:1903708 | positive regulation of hemopoiesis | 9.92E-04 | 17 |
| BP | GO:0070227 | lymphocyte apoptotic process | 9.92E-04 | 11 |
| BP | GO:0007599 | hemostasis | 1.00E-03 | 21 |
| BP | GO:0002478 | antigen processing and presentation of exogenous peptide antigen | 1.04E-03 | 8 |
| BP | GO:0032930 | positive regulation of superoxide anion generation | 1.12E-03 | 6 |
| BP | GO:0001776 | leukocyte homeostasis | 1.25E-03 | 12 |
| BP | GO:0051209 | release of sequestered calcium ion into cytosol | 1.26E-03 | 14 |
| BP | GO:0031349 | positive regulation of defense response | 1.26E-03 | 24 |
| BP | GO:0006000 | fructose metabolic process | 1.26E-03 | 5 |
| BP | GO:0045058 | T cell selection | 1.26E-03 | 9 |
| BP | GO:0002260 | lymphocyte homeostasis | 1.30E-03 | 10 |
| BP | GO:0048002 | antigen processing and presentation of peptide antigen | 1.30E-03 | 10 |
| BP | GO:0051283 | negative regulation of sequestering of calcium ion | 1.34E-03 | 14 |
| BP | GO:0042113 | B cell activation | 1.34E-03 | 27 |
| BP | GO:0010466 | negative regulation of peptidase activity | 1.34E-03 | 23 |
| BP | GO:0006066 | alcohol metabolic process | 1.37E-03 | 28 |
| BP | GO:0060759 | regulation of response to cytokine stimulus | 1.37E-03 | 17 |
| BP | GO:0019370 | leukotriene biosynthetic process | 1.43E-03 | 6 |
| BP | GO:0070269 | pyroptosis | 1.43E-03 | 6 |
| BP | GO:0019886 | antigen processing and presentation of exogenous peptide antigen via MHC class II | 1.44E-03 | 7 |
| BP | GO:0006006 | glucose metabolic process | 1.51E-03 | 19 |
| BP | GO:0071674 | mononuclear cell migration | 1.51E-03 | 19 |
| BP | GO:0018108 | peptidyl-tyrosine phosphorylation | 1.53E-03 | 29 |
| BP | GO:0051282 | regulation of sequestering of calcium ion | 1.53E-03 | 14 |
| BP | GO:0002700 | regulation of production of molecular mediator of immune response | 1.53E-03 | 17 |
| BP | GO:0050792 | regulation of viral process | 1.53E-03 | 17 |
| BP | GO:0045621 | positive regulation of lymphocyte differentiation | 1.60E-03 | 13 |
| BP | GO:0006749 | glutathione metabolic process | 1.60E-03 | 10 |
| BP | GO:0030888 | regulation of B cell proliferation | 1.60E-03 | 10 |
| BP | GO:0006790 | sulfur compound metabolic process | 1.61E-03 | 27 |
| BP | GO:0050856 | regulation of T cell receptor signaling pathway | 1.64E-03 | 8 |
| BP | GO:0045059 | positive thymic T cell selection | 1.70E-03 | 5 |
| BP | GO:0018212 | peptidyl-tyrosine modification | 1.70E-03 | 29 |
| BP | GO:0001782 | B cell homeostasis | 1.70E-03 | 7 |
| BP | GO:0045582 | positive regulation of T cell differentiation | 1.71E-03 | 12 |
| BP | GO:0002720 | positive regulation of cytokine production involved in immune response | 1.77E-03 | 10 |
| BP | GO:0019319 | hexose biosynthetic process | 1.80E-03 | 11 |
| BP | GO:0034767 | positive regulation of ion transmembrane transport | 1.83E-03 | 17 |
| BP | GO:0007596 | blood coagulation | 1.84E-03 | 20 |
| BP | GO:0071887 | leukocyte apoptotic process | 1.84E-03 | 13 |
| BP | GO:0098754 | detoxification | 1.94E-03 | 16 |
| BP | GO:0035456 | response to interferon-beta | 2.03E-03 | 7 |
| BP | GO:0042219 | cellular modified amino acid catabolic process | 2.03E-03 | 7 |
| BP | GO:0070228 | regulation of lymphocyte apoptotic process | 2.04E-03 | 9 |
| BP | GO:0051208 | sequestering of calcium ion | 2.04E-03 | 14 |
| BP | GO:0051924 | regulation of calcium ion transport | 2.17E-03 | 22 |
| BP | GO:0050727 | regulation of inflammatory response | 2.31E-03 | 29 |
| BP | GO:0046459 | short-chain fatty acid metabolic process | 2.33E-03 | 5 |
| BP | GO:0050817 | coagulation | 2.41E-03 | 20 |
| BP | GO:2000106 | regulation of leukocyte apoptotic process | 2.41E-03 | 11 |
| BP | GO:0002711 | positive regulation of T cell mediated immunity | 2.65E-03 | 9 |
| BP | GO:0043648 | dicarboxylic acid metabolic process | 2.68E-03 | 12 |
| BP | GO:0030183 | B cell differentiation | 2.68E-03 | 15 |
| BP | GO:0097553 | calcium ion transmembrane import into cytosol | 2.89E-03 | 15 |
| BP | GO:0002495 | antigen processing and presentation of peptide antigen via MHC class II | 2.90E-03 | 7 |
| BP | GO:0050869 | negative regulation of B cell activation | 2.90E-03 | 7 |
| BP | GO:0044270 | cellular nitrogen compound catabolic process | 2.92E-03 | 32 |
| BP | GO:0019058 | viral life cycle | 3.05E-03 | 25 |
| BP | GO:0002548 | monocyte chemotaxis | 3.06E-03 | 10 |
| BP | GO:0009074 | aromatic amino acid family catabolic process | 3.12E-03 | 5 |
| BP | GO:2001267 | regulation of cysteine-type endopeptidase activity involved in apoptotic signaling pathway | 3.12E-03 | 5 |
| BP | GO:0002685 | regulation of leukocyte migration | 3.17E-03 | 19 |
| BP | GO:0032612 | interleukin-1 production | 3.17E-03 | 14 |
| BP | GO:0032652 | regulation of interleukin-1 production | 3.17E-03 | 14 |
| BP | GO:0050731 | positive regulation of peptidyl-tyrosine phosphorylation | 3.17E-03 | 18 |
| BP | GO:0060402 | calcium ion transport into cytosol | 3.21E-03 | 16 |
| BP | GO:0016032 | viral process | 3.24E-03 | 30 |
| BP | GO:0043300 | regulation of leukocyte degranulation | 3.29E-03 | 8 |
| BP | GO:0002444 | myeloid leukocyte mediated immunity | 3.38E-03 | 12 |
| BP | GO:0033209 | tumor necrosis factor-mediated signaling pathway | 3.38E-03 | 12 |
| BP | GO:0009062 | fatty acid catabolic process | 3.70E-03 | 12 |
| BP | GO:2000117 | negative regulation of cysteine-type endopeptidase activity | 3.81E-03 | 11 |
| BP | GO:0044242 | cellular lipid catabolic process | 3.90E-03 | 19 |
| BP | GO:0002504 | antigen processing and presentation of peptide or polysaccharide antigen via MHC class II | 3.96E-03 | 7 |
| BP | GO:0042102 | positive regulation of T cell proliferation | 4.01E-03 | 12 |
| BP | GO:0002407 | dendritic cell chemotaxis | 4.13E-03 | 6 |
| BP | GO:0042060 | wound healing | 4.16E-03 | 30 |
| BP | GO:0097696 | receptor signaling pathway via STAT | 4.16E-03 | 17 |
| BP | GO:1901568 | fatty acid derivative metabolic process | 4.52E-03 | 10 |
| BP | GO:0051235 | maintenance of location | 4.52E-03 | 25 |
| BP | GO:0043368 | positive T cell selection | 4.54E-03 | 7 |
| BP | GO:0001915 | negative regulation of T cell mediated cytotoxicity | 4.54E-03 | 4 |
| BP | GO:1901748 | leukotriene D4 metabolic process | 4.54E-03 | 4 |
| BP | GO:1901750 | leukotriene D4 biosynthetic process | 4.54E-03 | 4 |
| BP | GO:1902237 | positive regulation of endoplasmic reticulum stress-induced intrinsic apoptotic signaling pathway | 4.54E-03 | 4 |
| BP | GO:2001269 | positive regulation of cysteine-type endopeptidase activity involved in apoptotic signaling pathway | 4.54E-03 | 4 |
| BP | GO:0046700 | heterocycle catabolic process | 4.54E-03 | 31 |
| BP | GO:0048872 | homeostasis of number of cells | 4.65E-03 | 22 |
| BP | GO:0019439 | aromatic compound catabolic process | 4.84E-03 | 32 |
| BP | GO:0006094 | gluconeogenesis | 4.85E-03 | 10 |
| BP | GO:0007263 | nitric oxide mediated signal transduction | 4.85E-03 | 6 |
| BP | GO:0032703 | negative regulation of interleukin-2 production | 4.85E-03 | 6 |
| BP | GO:0036037 | CD8-positive, alpha-beta T cell activation | 4.85E-03 | 6 |
| BP | GO:0034764 | positive regulation of transmembrane transport | 4.86E-03 | 19 |
| BP | GO:0032623 | interleukin-2 production | 4.96E-03 | 9 |
| BP | GO:0032663 | regulation of interleukin-2 production | 4.96E-03 | 9 |
| BP | GO:0045576 | mast cell activation | 4.96E-03 | 9 |
| BP | GO:0002283 | neutrophil activation involved in immune response | 5.06E-03 | 5 |
| BP | GO:0019932 | second-messenger-mediated signaling | 5.10E-03 | 24 |
| BP | GO:0009595 | detection of biotic stimulus | 5.14E-03 | 7 |
| BP | GO:0070664 | negative regulation of leukocyte proliferation | 5.16E-03 | 11 |
| BP | GO:0008652 | cellular amino acid biosynthetic process | 5.20E-03 | 10 |
| BP | GO:2000379 | positive regulation of reactive oxygen species metabolic process | 5.20E-03 | 10 |
| BP | GO:0001961 | positive regulation of cytokine-mediated signaling pathway | 5.26E-03 | 8 |
| BP | GO:0070231 | T cell apoptotic process | 5.26E-03 | 8 |
| BP | GO:0072593 | reactive oxygen species metabolic process | 5.26E-03 | 20 |
| BP | GO:0002704 | negative regulation of leukocyte mediated immunity | 5.41E-03 | 9 |
| BP | GO:0070059 | intrinsic apoptotic signaling pathway in response to endoplasmic reticulum stress | 5.41E-03 | 9 |
| BP | GO:0045862 | positive regulation of proteolysis | 5.44E-03 | 27 |
| BP | GO:0007259 | receptor signaling pathway via JAK-STAT | 5.50E-03 | 16 |
| BP | GO:0062012 | regulation of small molecule metabolic process | 5.60E-03 | 25 |
| BP | GO:0010818 | T cell chemotaxis | 5.62E-03 | 6 |
| BP | GO:0002688 | regulation of leukocyte chemotaxis | 5.82E-03 | 13 |
| BP | GO:0051341 | regulation of oxidoreductase activity | 6.03E-03 | 12 |
| BP | GO:0006919 | activation of cysteine-type endopeptidase activity involved in apoptotic process | 6.17E-03 | 10 |
| BP | GO:0043154 | negative regulation of cysteine-type endopeptidase activity involved in apoptotic process | 6.17E-03 | 10 |
| BP | GO:0033194 | response to hydroperoxide | 6.23E-03 | 5 |
| BP | GO:0006527 | arginine catabolic process | 6.23E-03 | 4 |
| BP | GO:0006751 | glutathione catabolic process | 6.23E-03 | 4 |
| BP | GO:0002832 | negative regulation of response to biotic stimulus | 6.44E-03 | 12 |
| BP | GO:0009069 | serine family amino acid metabolic process | 6.65E-03 | 7 |
| BP | GO:0051281 | positive regulation of release of sequestered calcium ion into cytosol | 6.65E-03 | 7 |
| BP | GO:0001916 | positive regulation of T cell mediated cytotoxicity | 6.65E-03 | 6 |
| BP | GO:0007584 | response to nutrient | 6.82E-03 | 16 |
| BP | GO:0032102 | negative regulation of response to external stimulus | 7.21E-03 | 29 |
| BP | GO:0009072 | aromatic amino acid family metabolic process | 7.94E-03 | 6 |
| BP | GO:0019674 | NAD metabolic process | 7.94E-03 | 6 |
| BP | GO:0050858 | negative regulation of antigen receptor-mediated signaling pathway | 7.94E-03 | 6 |
| BP | GO:0071260 | cellular response to mechanical stimulus | 8.05E-03 | 10 |
| BP | GO:0098739 | import across plasma membrane | 8.09E-03 | 16 |
| BP | GO:0009176 | pyrimidine deoxyribonucleoside monophosphate metabolic process | 8.66E-03 | 4 |
| BP | GO:0043383 | negative T cell selection | 8.66E-03 | 4 |
| BP | GO:0046185 | aldehyde catabolic process | 8.66E-03 | 4 |
| BP | GO:0060907 | positive regulation of macrophage cytokine production | 8.66E-03 | 4 |
| BP | GO:0071635 | negative regulation of transforming growth factor beta production | 8.66E-03 | 4 |
| BP | GO:0072330 | monocarboxylic acid biosynthetic process | 8.77E-03 | 18 |
| BP | GO:0010959 | regulation of metal ion transport | 8.78E-03 | 28 |
| BP | GO:0046686 | response to cadmium ion | 8.78E-03 | 9 |
| BP | GO:0002708 | positive regulation of lymphocyte mediated immunity | 9.19E-03 | 12 |
| BP | GO:0050672 | negative regulation of lymphocyte proliferation | 9.47E-03 | 10 |
| BP | GO:0002689 | negative regulation of leukocyte chemotaxis | 9.62E-03 | 5 |
| BP | GO:0002286 | T cell activation involved in immune response | 9.86E-03 | 12 |
| BP | GO:0070371 | ERK1 and ERK2 cascade | 9.93E-03 | 24 |
| BP | GO:0070374 | positive regulation of ERK1 and ERK2 cascade | 1.01E-02 | 18 |
| BP | GO:0032945 | negative regulation of mononuclear cell proliferation | 1.02E-02 | 10 |
| BP | GO:0071214 | cellular response to abiotic stimulus | 1.02E-02 | 24 |
| BP | GO:0104004 | cellular response to environmental stimulus | 1.02E-02 | 24 |
| BP | GO:0045861 | negative regulation of proteolysis | 1.03E-02 | 25 |
| BP | GO:0060401 | cytosolic calcium ion transport | 1.04E-02 | 16 |
| BP | GO:1903900 | regulation of viral life cycle | 1.06E-02 | 14 |
| BP | GO:0009262 | deoxyribonucleotide metabolic process | 1.11E-02 | 7 |
| BP | GO:0015749 | monosaccharide transmembrane transport | 1.12E-02 | 12 |
| BP | GO:1990748 | cellular detoxification | 1.12E-02 | 12 |
| BP | GO:0060760 | positive regulation of response to cytokine stimulus | 1.12E-02 | 8 |
| BP | GO:0006753 | nucleoside phosphate metabolic process | 1.14E-02 | 32 |
| BP | GO:0046415 | urate metabolic process | 1.14E-02 | 4 |
| BP | GO:0045061 | thymic T cell selection | 1.15E-02 | 5 |
| BP | GO:0050860 | negative regulation of T cell receptor signaling pathway | 1.15E-02 | 5 |
| BP | GO:0098869 | cellular oxidant detoxification | 1.16E-02 | 11 |
| BP | GO:0031348 | negative regulation of defense response | 1.18E-02 | 20 |
| BP | GO:0002702 | positive regulation of production of molecular mediator of immune response | 1.18E-02 | 12 |
| BP | GO:0002335 | mature B cell differentiation | 1.22E-02 | 6 |
| BP | GO:0002431 | Fc receptor mediated stimulatory signaling pathway | 1.22E-02 | 6 |
| BP | GO:0006536 | glutamate metabolic process | 1.22E-02 | 6 |
| BP | GO:0036336 | dendritic cell migration | 1.22E-02 | 6 |
| BP | GO:1904064 | positive regulation of cation transmembrane transport | 1.23E-02 | 14 |
| BP | GO:1904062 | regulation of cation transmembrane transport | 1.25E-02 | 25 |
| BP | GO:0034219 | carbohydrate transmembrane transport | 1.25E-02 | 12 |
| BP | GO:0008643 | carbohydrate transport | 1.31E-02 | 14 |
| BP | GO:0019395 | fatty acid oxidation | 1.33E-02 | 11 |
| BP | GO:0050848 | regulation of calcium-mediated signaling | 1.35E-02 | 9 |
| BP | GO:0042398 | cellular modified amino acid biosynthetic process | 1.38E-02 | 7 |
| BP | GO:0098751 | bone cell development | 1.40E-02 | 6 |
| BP | GO:0016485 | protein processing | 1.41E-02 | 18 |
| BP | GO:0016052 | carbohydrate catabolic process | 1.46E-02 | 14 |
| BP | GO:0009159 | deoxyribonucleoside monophosphate catabolic process | 1.47E-02 | 4 |
| BP | GO:0009173 | pyrimidine ribonucleoside monophosphate metabolic process | 1.47E-02 | 4 |
| BP | GO:0046049 | UMP metabolic process | 1.47E-02 | 4 |
| BP | GO:0031663 | lipopolysaccharide-mediated signaling pathway | 1.48E-02 | 8 |
| BP | GO:0046434 | organophosphate catabolic process | 1.53E-02 | 14 |
| BP | GO:1905898 | positive regulation of response to endoplasmic reticulum stress | 1.60E-02 | 6 |
| BP | GO:0050868 | negative regulation of T cell activation | 1.60E-02 | 12 |
| BP | GO:0051928 | positive regulation of calcium ion transport | 1.60E-02 | 12 |
| BP | GO:0009117 | nucleotide metabolic process | 1.61E-02 | 31 |
| BP | GO:0043030 | regulation of macrophage activation | 1.61E-02 | 8 |
| BP | GO:2000401 | regulation of lymphocyte migration | 1.61E-02 | 8 |
| BP | GO:0006817 | phosphate ion transport | 1.61E-02 | 5 |
| BP | GO:0009219 | pyrimidine deoxyribonucleotide metabolic process | 1.61E-02 | 5 |
| BP | GO:0032528 | microvillus organization | 1.61E-02 | 5 |
| BP | GO:0035458 | cellular response to interferon-beta | 1.61E-02 | 5 |
| BP | GO:2000377 | regulation of reactive oxygen species metabolic process | 1.68E-02 | 14 |
| BP | GO:0002686 | negative regulation of leukocyte migration | 1.69E-02 | 7 |
| BP | GO:0034765 | regulation of ion transmembrane transport | 1.69E-02 | 31 |
| BP | GO:0051051 | negative regulation of transport | 1.69E-02 | 30 |
| BP | GO:0007229 | integrin-mediated signaling pathway | 1.71E-02 | 11 |
| BP | GO:0043122 | regulation of I-kappaB kinase/NF-kappaB signaling | 1.71E-02 | 19 |
| BP | GO:0097193 | intrinsic apoptotic signaling pathway | 1.74E-02 | 21 |
| BP | GO:0032615 | interleukin-12 production | 1.75E-02 | 8 |
| BP | GO:0032655 | regulation of interleukin-12 production | 1.75E-02 | 8 |
| BP | GO:0043087 | regulation of GTPase activity | 1.76E-02 | 24 |
| BP | GO:0097237 | cellular response to toxic substance | 1.76E-02 | 12 |
| BP | GO:0002285 | lymphocyte activation involved in immune response | 1.77E-02 | 16 |
| BP | GO:1903038 | negative regulation of leukocyte cell-cell adhesion | 1.77E-02 | 13 |
| BP | GO:0048525 | negative regulation of viral process | 1.78E-02 | 10 |
| BP | GO:0034440 | lipid oxidation | 1.80E-02 | 11 |
| BP | GO:0070372 | regulation of ERK1 and ERK2 cascade | 1.80E-02 | 22 |
| BP | GO:0002468 | dendritic cell antigen processing and presentation | 1.80E-02 | 4 |
| BP | GO:0006570 | tyrosine metabolic process | 1.80E-02 | 4 |
| BP | GO:0035435 | phosphate ion transmembrane transport | 1.80E-02 | 4 |
| BP | GO:1903169 | regulation of calcium ion transmembrane transport | 1.81E-02 | 14 |
| BP | GO:0044403 | biological process involved in symbiotic interaction | 1.83E-02 | 21 |
| BP | GO:0098581 | detection of external biotic stimulus | 1.85E-02 | 5 |
| BP | GO:0032755 | positive regulation of interleukin-6 production | 1.89E-02 | 10 |
| BP | GO:0002369 | T cell cytokine production | 1.99E-02 | 6 |
| BP | GO:0002724 | regulation of T cell cytokine production | 1.99E-02 | 6 |
| BP | GO:0072529 | pyrimidine-containing compound catabolic process | 1.99E-02 | 6 |
| BP | GO:0002698 | negative regulation of immune effector process | 2.01E-02 | 11 |
| BP | GO:1904659 | glucose transmembrane transport | 2.01E-02 | 11 |
| BP | GO:0038093 | Fc receptor signaling pathway | 2.01E-02 | 7 |
| BP | GO:0060337 | type I interferon signaling pathway | 2.01E-02 | 7 |
| BP | GO:1901570 | fatty acid derivative biosynthetic process | 2.01E-02 | 7 |
| BP | GO:0046718 | viral entry into host cell | 2.04E-02 | 13 |
| BP | GO:0051279 | regulation of release of sequestered calcium ion into cytosol | 2.08E-02 | 9 |
| BP | GO:0002710 | negative regulation of T cell mediated immunity | 2.14E-02 | 5 |
| BP | GO:0009164 | nucleoside catabolic process | 2.14E-02 | 5 |
| BP | GO:0019883 | antigen processing and presentation of endogenous antigen | 2.14E-02 | 5 |
| BP | GO:0050857 | positive regulation of antigen receptor-mediated signaling pathway | 2.14E-02 | 5 |
| BP | GO:0043410 | positive regulation of MAPK cascade | 2.14E-02 | 30 |
| BP | GO:0002830 | positive regulation of type 2 immune response | 2.19E-02 | 4 |
| BP | GO:0006544 | glycine metabolic process | 2.19E-02 | 4 |
| BP | GO:0006750 | glutathione biosynthetic process | 2.19E-02 | 4 |
| BP | GO:0010819 | regulation of T cell chemotaxis | 2.19E-02 | 4 |
| BP | GO:0046135 | pyrimidine nucleoside catabolic process | 2.19E-02 | 4 |
| BP | GO:0046633 | alpha-beta T cell proliferation | 2.19E-02 | 6 |
| BP | GO:0097242 | amyloid-beta clearance | 2.19E-02 | 6 |
| BP | GO:0001676 | long-chain fatty acid metabolic process | 2.24E-02 | 11 |
| BP | GO:0001818 | negative regulation of cytokine production | 2.26E-02 | 24 |
| BP | GO:0009410 | response to xenobiotic stimulus | 2.32E-02 | 29 |
| BP | GO:0044106 | cellular amine metabolic process | 2.38E-02 | 11 |
| BP | GO:0072678 | T cell migration | 2.38E-02 | 8 |
| BP | GO:0002218 | activation of innate immune response | 2.40E-02 | 7 |
| BP | GO:0043090 | amino acid import | 2.40E-02 | 7 |
| BP | GO:0043277 | apoptotic cell clearance | 2.40E-02 | 7 |
| BP | GO:0071357 | cellular response to type I interferon | 2.40E-02 | 7 |
| BP | GO:0016051 | carbohydrate biosynthetic process | 2.41E-02 | 16 |
| BP | GO:0019722 | calcium-mediated signaling | 2.41E-02 | 16 |
| BP | GO:0008645 | hexose transmembrane transport | 2.50E-02 | 11 |
| BP | GO:0019751 | polyol metabolic process | 2.50E-02 | 11 |
| BP | GO:0050853 | B cell receptor signaling pathway | 2.51E-02 | 12 |
| BP | GO:0000302 | response to reactive oxygen species | 2.53E-02 | 17 |
| BP | GO:0007249 | I-kappaB kinase/NF-kappaB signaling | 2.61E-02 | 20 |
| BP | GO:0002707 | negative regulation of lymphocyte mediated immunity | 2.64E-02 | 7 |
| BP | GO:0010934 | macrophage cytokine production | 2.66E-02 | 4 |
| BP | GO:0010935 | regulation of macrophage cytokine production | 2.66E-02 | 4 |
| BP | GO:0070206 | protein trimerization | 2.66E-02 | 4 |
| BP | GO:0009394 | 2'-deoxyribonucleotide metabolic process | 2.73E-02 | 6 |
| BP | GO:0010543 | regulation of platelet activation | 2.73E-02 | 6 |
| BP | GO:0032892 | positive regulation of organic acid transport | 2.73E-02 | 6 |
| BP | GO:1901607 | alpha-amino acid biosynthetic process | 2.76E-02 | 8 |
| BP | GO:0007162 | negative regulation of cell adhesion | 2.78E-02 | 21 |
| BP | GO:0033559 | unsaturated fatty acid metabolic process | 2.78E-02 | 11 |
| BP | GO:0031342 | negative regulation of cell killing | 2.78E-02 | 5 |
| BP | GO:0038094 | Fc-gamma receptor signaling pathway | 2.78E-02 | 5 |
| BP | GO:0045589 | regulation of regulatory T cell differentiation | 2.78E-02 | 5 |
| BP | GO:0061082 | myeloid leukocyte cytokine production | 2.78E-02 | 5 |
| BP | GO:0044409 | entry into host | 2.82E-02 | 13 |
| BP | GO:0010524 | positive regulation of calcium ion transport into cytosol | 2.84E-02 | 7 |
| BP | GO:0072348 | sulfur compound transport | 2.84E-02 | 7 |
| BP | GO:0006814 | sodium ion transport | 2.88E-02 | 18 |
| BP | GO:0098657 | import into cell | 3.05E-02 | 17 |
| BP | GO:0051048 | negative regulation of secretion | 3.10E-02 | 14 |
| BP | GO:0009308 | amine metabolic process | 3.11E-02 | 11 |
| BP | GO:0031667 | response to nutrient levels | 3.11E-02 | 29 |
| BP | GO:0009068 | aspartate family amino acid catabolic process | 3.16E-02 | 4 |
| BP | GO:0009125 | nucleoside monophosphate catabolic process | 3.16E-02 | 4 |
| BP | GO:0019184 | nonribosomal peptide biosynthetic process | 3.16E-02 | 4 |
| BP | GO:0030889 | negative regulation of B cell proliferation | 3.16E-02 | 4 |
| BP | GO:0071800 | podosome assembly | 3.16E-02 | 4 |
| BP | GO:0009310 | amine catabolic process | 3.16E-02 | 5 |
| BP | GO:0090025 | regulation of monocyte chemotaxis | 3.16E-02 | 5 |
| BP | GO:1901136 | carbohydrate derivative catabolic process | 3.20E-02 | 14 |
| BP | GO:0002440 | production of molecular mediator of immune response | 3.20E-02 | 21 |
| BP | GO:0010522 | regulation of calcium ion transport into cytosol | 3.20E-02 | 10 |
| BP | GO:0034308 | primary alcohol metabolic process | 3.20E-02 | 10 |
| BP | GO:0062014 | negative regulation of small molecule metabolic process | 3.20E-02 | 10 |
| BP | GO:0008360 | regulation of cell shape | 3.20E-02 | 13 |
| BP | GO:0015908 | fatty acid transport | 3.25E-02 | 9 |
| BP | GO:0019692 | deoxyribose phosphate metabolic process | 3.28E-02 | 6 |
| BP | GO:0030890 | positive regulation of B cell proliferation | 3.28E-02 | 6 |
| BP | GO:0033003 | regulation of mast cell activation | 3.28E-02 | 6 |
| BP | GO:0061515 | myeloid cell development | 3.39E-02 | 8 |
| BP | GO:0030258 | lipid modification | 3.53E-02 | 16 |
| BP | GO:0009264 | deoxyribonucleotide catabolic process | 3.58E-02 | 5 |
| BP | GO:0002224 | toll-like receptor signaling pathway | 3.60E-02 | 11 |
| BP | GO:0046634 | regulation of alpha-beta T cell activation | 3.60E-02 | 10 |
| BP | GO:0048661 | positive regulation of smooth muscle cell proliferation | 3.60E-02 | 10 |
| BP | GO:0052126 | movement in host environment | 3.62E-02 | 14 |
| BP | GO:1903793 | positive regulation of anion transport | 3.64E-02 | 6 |
| BP | GO:1903036 | positive regulation of response to wounding | 3.64E-02 | 8 |
| BP | GO:0002888 | positive regulation of myeloid leukocyte mediated immunity | 3.67E-02 | 4 |
| BP | GO:0009081 | branched-chain amino acid metabolic process | 3.67E-02 | 4 |
| BP | GO:0009223 | pyrimidine deoxyribonucleotide catabolic process | 3.67E-02 | 4 |
| BP | GO:0032695 | negative regulation of interleukin-12 production | 3.67E-02 | 4 |
| BP | GO:0043031 | negative regulation of macrophage activation | 3.67E-02 | 4 |
| BP | GO:0044546 | NLRP3 inflammasome complex assembly | 3.67E-02 | 4 |
| BP | GO:0048245 | eosinophil chemotaxis | 3.67E-02 | 4 |
| BP | GO:0140131 | positive regulation of lymphocyte chemotaxis | 3.67E-02 | 4 |
| BP | GO:2001185 | regulation of CD8-positive, alpha-beta T cell activation | 3.67E-02 | 4 |
| BP | GO:0051604 | protein maturation | 3.86E-02 | 20 |
| BP | GO:1904427 | positive regulation of calcium ion transmembrane transport | 3.87E-02 | 8 |
| BP | GO:0034340 | response to type I interferon | 3.90E-02 | 7 |
| BP | GO:2001244 | positive regulation of intrinsic apoptotic signaling pathway | 3.90E-02 | 7 |
| BP | GO:0022408 | negative regulation of cell-cell adhesion | 3.91E-02 | 15 |
| BP | GO:0031295 | T cell costimulation | 3.92E-02 | 6 |
| BP | GO:0150076 | neuroinflammatory response | 3.92E-02 | 6 |
| BP | GO:0002828 | regulation of type 2 immune response | 3.92E-02 | 5 |
| BP | GO:0034656 | nucleobase-containing small molecule catabolic process | 3.92E-02 | 5 |
| BP | GO:0043171 | peptide catabolic process | 3.92E-02 | 5 |
| BP | GO:0045066 | regulatory T cell differentiation | 3.92E-02 | 5 |
| BP | GO:0046386 | deoxyribose phosphate catabolic process | 3.92E-02 | 5 |
| BP | GO:0006090 | pyruvate metabolic process | 3.92E-02 | 10 |
| BP | GO:0046822 | regulation of nucleocytoplasmic transport | 3.92E-02 | 10 |
| BP | GO:0000050 | urea cycle | 3.93E-02 | 3 |
| BP | GO:0002765 | immune response-inhibiting signal transduction | 3.93E-02 | 3 |
| BP | GO:0002887 | negative regulation of myeloid leukocyte mediated immunity | 3.93E-02 | 3 |
| BP | GO:0006560 | proline metabolic process | 3.93E-02 | 3 |
| BP | GO:0006646 | phosphatidylethanolamine biosynthetic process | 3.93E-02 | 3 |
| BP | GO:0030388 | fructose 1,6-bisphosphate metabolic process | 3.93E-02 | 3 |
| BP | GO:0051918 | negative regulation of fibrinolysis | 3.93E-02 | 3 |
| BP | GO:0060368 | regulation of Fc receptor mediated stimulatory signaling pathway | 3.93E-02 | 3 |
| BP | GO:0006959 | humoral immune response | 4.06E-02 | 21 |
| BP | GO:0002820 | negative regulation of adaptive immune response | 4.12E-02 | 7 |
| BP | GO:0019369 | arachidonic acid metabolic process | 4.12E-02 | 7 |
| BP | GO:0090303 | positive regulation of wound healing | 4.12E-02 | 7 |
| BP | GO:0050864 | regulation of B cell activation | 4.12E-02 | 15 |
| BP | GO:0009129 | pyrimidine nucleoside monophosphate metabolic process | 4.20E-02 | 4 |
| BP | GO:0046629 | gamma-delta T cell activation | 4.20E-02 | 4 |
| BP | GO:0098543 | detection of other organism | 4.20E-02 | 4 |
| BP | GO:0140632 | inflammasome complex assembly | 4.20E-02 | 4 |
| BP | GO:0070555 | response to interleukin-1 | 4.26E-02 | 12 |
| BP | GO:0060191 | regulation of lipase activity | 4.26E-02 | 9 |
| BP | GO:0033077 | T cell differentiation in thymus | 4.30E-02 | 8 |
| BP | GO:0072337 | modified amino acid transport | 4.33E-02 | 5 |
| BP | GO:1902235 | regulation of endoplasmic reticulum stress-induced intrinsic apoptotic signaling pathway | 4.33E-02 | 5 |
| BP | GO:0071496 | cellular response to external stimulus | 4.40E-02 | 21 |
| BP | GO:0006081 | cellular aldehyde metabolic process | 4.41E-02 | 7 |
| BP | GO:0031638 | zymogen activation | 4.41E-02 | 7 |
| BP | GO:0051851 | modulation by host of symbiont process | 4.41E-02 | 7 |
| BP | GO:2001235 | positive regulation of apoptotic signaling pathway | 4.42E-02 | 11 |
| BP | GO:0072332 | intrinsic apoptotic signaling pathway by p53 class mediator | 4.60E-02 | 8 |
| BP | GO:0031294 | lymphocyte costimulation | 4.62E-02 | 6 |
| BP | GO:2000107 | negative regulation of leukocyte apoptotic process | 4.62E-02 | 6 |
| BP | GO:0009259 | ribonucleotide metabolic process | 4.63E-02 | 24 |
| BP | GO:0006067 | ethanol metabolic process | 4.88E-02 | 4 |
| BP | GO:0006244 | pyrimidine nucleotide catabolic process | 4.88E-02 | 4 |
| BP | GO:0051767 | nitric-oxide synthase biosynthetic process | 4.88E-02 | 4 |
| BP | GO:0051769 | regulation of nitric-oxide synthase biosynthetic process | 4.88E-02 | 4 |
| BP | GO:0061081 | positive regulation of myeloid leukocyte cytokine production involved in immune response | 4.88E-02 | 4 |
| BP | GO:0036490 | regulation of translation in response to endoplasmic reticulum stress | 4.92E-02 | 3 |
| BP | GO:0043312 | neutrophil degranulation | 4.92E-02 | 3 |
| BP | GO:0045060 | negative thymic T cell selection | 4.92E-02 | 3 |
| BP | GO:0070314 | G1 to G0 transition | 4.92E-02 | 3 |
| BP | GO:1903265 | positive regulation of tumor necrosis factor-mediated signaling pathway | 4.92E-02 | 3 |
| BP | GO:1903961 | positive regulation of anion transmembrane transport | 4.92E-02 | 3 |
| BP | GO:1905522 | negative regulation of macrophage migration | 4.92E-02 | 3 |
| BP | GO:0032635 | interleukin-6 production | 4.92E-02 | 13 |
| BP | GO:0032675 | regulation of interleukin-6 production | 4.92E-02 | 13 |
| BP | GO:0048659 | smooth muscle cell proliferation | 4.93E-02 | 14 |
| BP | GO:0050920 | regulation of chemotaxis | 4.95E-02 | 16 |
| BP | GO:0046677 | response to antibiotic | 4.97E-02 | 6 |
| BP | GO:0097300 | programmed necrotic cell death | 4.97E-02 | 6 |
| CC | GO:0016324 | apical plasma membrane | 2.48E-08 | 41 |
| CC | GO:0045177 | apical part of cell | 4.96E-08 | 44 |
| CC | GO:0009897 | external side of plasma membrane | 4.96E-08 | 43 |
| CC | GO:0005903 | brush border | 7.38E-08 | 20 |
| CC | GO:0031526 | brush border membrane | 8.14E-08 | 15 |
| CC | GO:0045121 | membrane raft | 1.82E-06 | 34 |
| CC | GO:0098857 | membrane microdomain | 1.82E-06 | 34 |
| CC | GO:0030667 | secretory granule membrane | 3.13E-05 | 30 |
| CC | GO:0098862 | cluster of actin-based cell projections | 4.59E-05 | 20 |
| CC | GO:0070820 | tertiary granule | 8.20E-04 | 18 |
| CC | GO:0042581 | specific granule | 1.92E-03 | 17 |
| CC | GO:0044853 | plasma membrane raft | 1.96E-03 | 14 |
| CC | GO:0005782 | peroxisomal matrix | 1.96E-03 | 9 |
| CC | GO:0031907 | microbody lumen | 1.96E-03 | 9 |
| CC | GO:0034774 | secretory granule lumen | 2.12E-03 | 26 |
| CC | GO:0060205 | cytoplasmic vesicle lumen | 2.28E-03 | 26 |
| CC | GO:0101002 | ficolin-1-rich granule | 2.28E-03 | 18 |
| CC | GO:0031983 | vesicle lumen | 2.28E-03 | 26 |
| CC | GO:0005775 | vacuolar lumen | 3.17E-03 | 17 |
| CC | GO:0030139 | endocytic vesicle | 3.18E-03 | 26 |
| CC | GO:0016323 | basolateral plasma membrane | 4.04E-03 | 20 |
| CC | GO:0009925 | basal plasma membrane | 5.96E-03 | 21 |
| CC | GO:0045335 | phagocytic vesicle | 6.32E-03 | 14 |
| CC | GO:0030666 | endocytic vesicle membrane | 8.60E-03 | 17 |
| CC | GO:1904724 | tertiary granule lumen | 1.11E-02 | 8 |
| CC | GO:0045178 | basal part of cell | 1.25E-02 | 21 |
| CC | GO:0030665 | clathrin-coated vesicle membrane | 1.25E-02 | 12 |
| CC | GO:0001772 | immunological synapse | 1.25E-02 | 7 |
| CC | GO:0030669 | clathrin-coated endocytic vesicle membrane | 1.38E-02 | 9 |
| CC | GO:0043020 | NADPH oxidase complex | 1.70E-02 | 4 |
| CC | GO:0031253 | cell projection membrane | 1.73E-02 | 24 |
| CC | GO:0005902 | microvillus | 1.82E-02 | 10 |
| CC | GO:1904813 | ficolin-1-rich granule lumen | 1.86E-02 | 12 |
| CC | GO:0042470 | melanosome | 1.96E-02 | 11 |
| CC | GO:0048770 | pigment granule | 1.96E-02 | 11 |
| CC | GO:0061702 | inflammasome complex | 3.10E-02 | 4 |
| CC | GO:0002102 | podosome | 3.74E-02 | 5 |
| CC | GO:0005765 | lysosomal membrane | 3.80E-02 | 25 |
| CC | GO:0098852 | lytic vacuole membrane | 3.80E-02 | 25 |
| CC | GO:0005925 | focal adhesion | 3.82E-02 | 26 |
| CC | GO:0005788 | endoplasmic reticulum lumen | 3.82E-02 | 21 |
| CC | GO:0070821 | tertiary granule membrane | 4.21E-02 | 8 |
| CC | GO:0008305 | integrin complex | 4.23E-02 | 5 |
| CC | GO:0005777 | peroxisome | 4.23E-02 | 12 |
| CC | GO:0042579 | microbody | 4.23E-02 | 12 |
| CC | GO:0030055 | cell-substrate junction | 4.23E-02 | 26 |
| CC | GO:0035579 | specific granule membrane | 4.28E-02 | 9 |
| CC | GO:0045334 | clathrin-coated endocytic vesicle | 4.28E-02 | 9 |
| CC | GO:0030662 | coated vesicle membrane | 4.30E-02 | 14 |
| CC | GO:0098858 | actin-based cell projection | 4.46E-02 | 16 |
| MF | GO:0015291 | secondary active transmembrane transporter activity | 1.05E-06 | 31 |
| MF | GO:0016614 | oxidoreductase activity, acting on CH-OH group of donors | 5.37E-05 | 20 |
| MF | GO:0042379 | chemokine receptor binding | 8.48E-05 | 14 |
| MF | GO:0022853 | active ion transmembrane transporter activity | 1.42E-04 | 28 |
| MF | GO:0008009 | chemokine activity | 2.11E-04 | 11 |
| MF | GO:0015293 | symporter activity | 7.07E-04 | 18 |
| MF | GO:0140375 | immune receptor activity | 7.07E-04 | 18 |
| MF | GO:0046943 | carboxylic acid transmembrane transporter activity | 9.08E-04 | 19 |
| MF | GO:0005342 | organic acid transmembrane transporter activity | 9.08E-04 | 19 |
| MF | GO:0016616 | oxidoreductase activity, acting on the CH-OH group of donors, NAD or NADP as acceptor | 9.08E-04 | 16 |
| MF | GO:0050660 | flavin adenine dinucleotide binding | 9.08E-04 | 13 |
| MF | GO:0015370 | solute:sodium symporter activity | 9.08E-04 | 12 |
| MF | GO:0005402 | carbohydrate:cation symporter activity | 9.08E-04 | 6 |
| MF | GO:0016597 | amino acid binding | 9.33E-04 | 10 |
| MF | GO:0008514 | organic anion transmembrane transporter activity | 9.33E-04 | 20 |
| MF | GO:0019864 | IgG binding | 1.33E-03 | 5 |
| MF | GO:0015294 | solute:cation symporter activity | 1.34E-03 | 14 |
| MF | GO:0023023 | MHC protein complex binding | 1.81E-03 | 8 |
| MF | GO:0022804 | active transmembrane transporter activity | 2.07E-03 | 32 |
| MF | GO:0048020 | CCR chemokine receptor binding | 2.27E-03 | 9 |
| MF | GO:0030246 | carbohydrate binding | 2.76E-03 | 24 |
| MF | GO:0015297 | antiporter activity | 3.78E-03 | 12 |
| MF | GO:0043177 | organic acid binding | 4.88E-03 | 15 |
| MF | GO:0019865 | immunoglobulin binding | 4.88E-03 | 6 |
| MF | GO:0004029 | aldehyde dehydrogenase (NAD+) activity | 4.88E-03 | 5 |
| MF | GO:0005126 | cytokine receptor binding | 5.71E-03 | 23 |
| MF | GO:0015145 | monosaccharide transmembrane transporter activity | 5.71E-03 | 6 |
| MF | GO:0004030 | aldehyde dehydrogenase [NAD(P)+] activity | 6.15E-03 | 5 |
| MF | GO:0001664 | G protein-coupled receptor binding | 7.31E-03 | 24 |
| MF | GO:0008509 | anion transmembrane transporter activity | 7.38E-03 | 25 |
| MF | GO:0016627 | oxidoreductase activity, acting on the CH-CH group of donors | 8.71E-03 | 9 |
| MF | GO:0061134 | peptidase regulator activity | 8.82E-03 | 20 |
| MF | GO:0004866 | endopeptidase inhibitor activity | 8.82E-03 | 17 |
| MF | GO:0004497 | monooxygenase activity | 8.82E-03 | 12 |
| MF | GO:0051119 | sugar transmembrane transporter activity | 8.82E-03 | 6 |
| MF | GO:0045236 | CXCR chemokine receptor binding | 8.82E-03 | 5 |
| MF | GO:0046906 | tetrapyrrole binding | 9.10E-03 | 15 |
| MF | GO:0042608 | T cell receptor binding | 1.07E-02 | 4 |
| MF | GO:0016628 | oxidoreductase activity, acting on the CH-CH group of donors, NAD or NADP as acceptor | 1.16E-02 | 6 |
| MF | GO:0030414 | peptidase inhibitor activity | 1.16E-02 | 17 |
| MF | GO:0015144 | carbohydrate transmembrane transporter activity | 1.16E-02 | 7 |
| MF | GO:0016810 | hydrolase activity, acting on carbon-nitrogen (but not peptide) bonds | 1.40E-02 | 13 |
| MF | GO:0061135 | endopeptidase regulator activity | 1.66E-02 | 17 |
| MF | GO:0016709 | oxidoreductase activity, acting on paired donors, with incorporation or reduction of molecular oxygen, NAD(P)H as one donor, and incorporation of one atom of oxygen | 1.70E-02 | 7 |
| MF | GO:0033218 | amide binding | 1.71E-02 | 28 |
| MF | GO:0005355 | glucose transmembrane transporter activity | 1.84E-02 | 5 |
| MF | GO:0015149 | hexose transmembrane transporter activity | 1.84E-02 | 5 |
| MF | GO:0140323 | solute:anion antiporter activity | 1.99E-02 | 6 |
| MF | GO:0008201 | heparin binding | 2.11E-02 | 15 |
| MF | GO:0008238 | exopeptidase activity | 2.39E-02 | 11 |
| MF | GO:0015081 | sodium ion transmembrane transporter activity | 2.43E-02 | 14 |
| MF | GO:0016701 | oxidoreductase activity, acting on single donors with incorporation of molecular oxygen | 2.47E-02 | 5 |
| MF | GO:0043274 | phospholipase binding | 2.47E-02 | 5 |
| MF | GO:1990782 | protein tyrosine kinase binding | 2.57E-02 | 11 |
| MF | GO:0043621 | protein self-association | 2.57E-02 | 8 |
| MF | GO:0001846 | opsonin binding | 2.61E-02 | 4 |
| MF | GO:0016805 | dipeptidase activity | 2.61E-02 | 4 |
| MF | GO:0020037 | heme binding | 2.71E-02 | 13 |
| MF | GO:1901681 | sulfur compound binding | 2.82E-02 | 20 |
| MF | GO:0016620 | oxidoreductase activity, acting on the aldehyde or oxo group of donors, NAD or NADP as acceptor | 2.95E-02 | 6 |
| MF | GO:0005539 | glycosaminoglycan binding | 3.02E-02 | 18 |
| MF | GO:0015556 | C4-dicarboxylate transmembrane transporter activity | 3.11E-02 | 4 |
| MF | GO:0008047 | enzyme activator activity | 3.21E-02 | 11 |
| MF | GO:0004175 | endopeptidase activity | 3.45E-02 | 28 |
| MF | GO:0016405 | CoA-ligase activity | 3.45E-02 | 5 |
| MF | GO:0023026 | MHC class II protein complex binding | 3.45E-02 | 5 |
| MF | GO:0005324 | long-chain fatty acid transporter activity | 3.65E-02 | 4 |
| MF | GO:0004896 | cytokine receptor activity | 3.69E-02 | 10 |
| MF | GO:0043028 | cysteine-type endopeptidase regulator activity involved in apoptotic process | 3.84E-02 | 6 |
| MF | GO:0051861 | glycolipid binding | 3.84E-02 | 5 |
| MF | GO:0016755 | aminoacyltransferase activity | 4.24E-02 | 4 |
| MF | GO:0060229 | lipase activator activity | 4.24E-02 | 4 |
| MF | GO:0015301 | anion:anion antiporter activity | 4.25E-02 | 5 |
| MF | GO:0016645 | oxidoreductase activity, acting on the CH-NH group of donors | 4.25E-02 | 5 |
| MF | GO:0016705 | oxidoreductase activity, acting on paired donors, with incorporation or reduction of molecular oxygen | 4.61E-02 | 14 |
| MF | GO:0002020 | protease binding | 4.61E-02 | 12 |
| MF | GO:0016209 | antioxidant activity | 4.68E-02 | 9 |
| MF | GO:0005310 | dicarboxylic acid transmembrane transporter activity | 4.70E-02 | 5 |
| MF | GO:0004197 | cysteine-type endopeptidase activity | 4.71E-02 | 11 |
| MF | GO:0008234 | cysteine-type peptidase activity | 4.77E-02 | 15 |

Supplementary Table 3. The details of GO enrichment analysis.

| ID | Description | p.adjust | Count |
| --- | --- | --- | --- |
| hsa05140 | Leishmaniasis | 1.02E-04 | 16 |
| hsa04062 | Chemokine signaling pathway | 3.72E-04 | 25 |
| hsa05152 | Tuberculosis | 8.67E-04 | 23 |
| hsa04061 | Viral protein interaction with cytokine and cytokine receptor | 9.40E-04 | 16 |
| hsa04640 | Hematopoietic cell lineage | 2.15E-03 | 15 |
| hsa04621 | NOD-like receptor signaling pathway | 2.15E-03 | 22 |
| hsa00410 | beta-Alanine metabolism | 2.15E-03 | 8 |
| hsa04670 | Leukocyte transendothelial migration | 2.15E-03 | 16 |
| hsa00260 | Glycine, serine and threonine metabolism | 2.15E-03 | 9 |
| hsa04145 | Phagosome | 2.15E-03 | 19 |
| hsa04666 | Fc gamma R-mediated phagocytosis | 3.80E-03 | 14 |
| hsa04668 | TNF signaling pathway | 5.83E-03 | 15 |
| hsa04658 | Th1 and Th2 cell differentiation | 6.87E-03 | 13 |
| hsa05417 | Lipid and atherosclerosis | 8.30E-03 | 22 |
| hsa04933 | AGE-RAGE signaling pathway in diabetic complications | 1.31E-02 | 13 |
| hsa05130 | Pathogenic Escherichia coli infection | 1.31E-02 | 20 |
| hsa04380 | Osteoclast differentiation | 1.31E-02 | 15 |
| hsa00120 | Primary bile acid biosynthesis | 1.31E-02 | 5 |
| hsa01200 | Carbon metabolism | 1.31E-02 | 14 |
| hsa04650 | Natural killer cell mediated cytotoxicity | 1.66E-02 | 15 |
| hsa05323 | Rheumatoid arthritis | 1.66E-02 | 12 |
| hsa00620 | Pyruvate metabolism | 1.75E-02 | 8 |
| hsa04659 | Th17 cell differentiation | 1.83E-02 | 13 |
| hsa04611 | Platelet activation | 2.18E-02 | 14 |
| hsa01230 | Biosynthesis of amino acids | 2.53E-02 | 10 |
| hsa03320 | PPAR signaling pathway | 2.53E-02 | 10 |
| hsa05164 | Influenza A | 2.53E-02 | 17 |
| hsa04060 | Cytokine-cytokine receptor interaction | 2.53E-02 | 25 |
| hsa04216 | Ferroptosis | 2.53E-02 | 7 |
| hsa05133 | Pertussis | 2.53E-02 | 10 |
| hsa05235 | PD-L1 expression and PD-1 checkpoint pathway in cancer | 2.53E-02 | 11 |
| hsa00220 | Arginine biosynthesis | 2.63E-02 | 5 |
| hsa00340 | Histidine metabolism | 2.63E-02 | 5 |
| hsa04064 | NF-kappa B signaling pathway | 2.69E-02 | 12 |
| hsa00010 | Glycolysis / Gluconeogenesis | 3.07E-02 | 9 |
| hsa00051 | Fructose and mannose metabolism | 3.07E-02 | 6 |
| hsa04210 | Apoptosis | 3.34E-02 | 14 |
| hsa05170 | Human immunodeficiency virus 1 infection | 3.34E-02 | 19 |
| hsa05135 | Yersinia infection | 3.34E-02 | 14 |
| hsa04146 | Peroxisome | 3.34E-02 | 10 |
| hsa05150 | Staphylococcus aureus infection | 3.46E-02 | 11 |
| hsa00240 | Pyrimidine metabolism | 3.59E-02 | 8 |
| hsa04662 | B cell receptor signaling pathway | 3.70E-02 | 10 |
| hsa05145 | Toxoplasmosis | 3.81E-02 | 12 |
| hsa04514 | Cell adhesion molecules | 4.08E-02 | 15 |
| hsa00250 | Alanine, aspartate and glutamate metabolism | 4.32E-02 | 6 |
| hsa00330 | Arginine and proline metabolism | 4.91E-02 | 7 |
| hsa05144 | Malaria | 4.91E-02 | 7 |

Supplementary Table 4. The details of KEGG enrichment analysis.
